# Advances in nanobiosensors during the COVID-19 pandemic and future perspectives for the post-COVID era

**DOI:** 10.1186/s40580-023-00410-5

**Published:** 2024-01-11

**Authors:** Young Jun Kim, Junhong Min

**Affiliations:** School of Integrative Engineering, Chung-Ang University, Heukseok-Dong, Dongjak-Gu, Seoul, 06974 Republic of Korea

**Keywords:** Nanobiosensors, Infectious virus, SARS-CoV-2, COVID-19 pandemic, Point-of-care testing

## Abstract

The unprecedented threat of the highly contagious virus, severe acute respiratory syndrome coronavirus 2 (SARS-CoV-2), which causes exponentially increased infections of coronavirus disease 2019 (COVID-19), highlights the weak spots of the current diagnostic toolbox. In the midst of catastrophe, nanobiosensors offer a new opportunity as an alternative tool to fill a gap among molecular tests, rapid antigen tests, and serological tests. Nanobiosensors surpass the potential of antigen tests because of their enhanced sensitivity, thus enabling us to see antigens as stable and easy-to-access targets. During the first three years of the COVID-19 pandemic, a substantial number of studies have reported nanobiosensors for the detection of SARS-CoV-2 antigens. The number of articles on nanobiosensors and SARS-CoV-2 exceeds the amount of nanobiosensor research on detecting previous infectious diseases, from influenza to SARS-CoV and MERS-CoV. This unprecedented publishing pace also implies the significance of SARS-CoV-2 and the present pandemic. In this review, 158 studies reporting nanobiosensors for detecting SARS-CoV-2 antigens are collected to discuss the current challenges of nanobiosensors using the criteria of point-of-care (POC) diagnostics along with COVID-specific issues. These advances and lessons during the pandemic pave the way for preparing for the post-COVID era and potential upcoming infectious diseases.

**Graphical Abstract:**

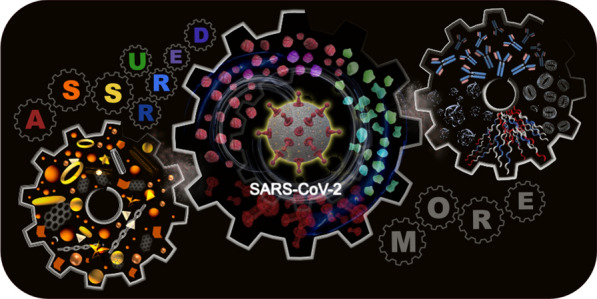

## Introduction

In December 2019, the first report of unidentified pneumonia cases in Wuhan, China, was disclosed [1]. These strange cases included symptoms such as fever, fatigue, anosmia, cough, headache, sore throat, chest discomfort, difficulty breathing, and loss of taste or smell [2, 3]. Some of those symptoms were very similar to symptoms of previous respiratory viral infections, such as influenza, severe acute respiratory syndrome-associated coronavirus (SARS-CoV), and Middle East respiratory syndrome-related coronavirus (MERS-CoV) [4, 5]. Because this new virus transmitted from person to person with high efficiency, it rapidly spread to all countries on earth and instantaneously paralyzed the public health system in most countries. The virus was named severe acute respiratory syndrome coronavirus 2 (SARS-CoV-2), and the World Health Organization (WHO) declared a pandemic on March 11, 2020. Until WTO declared the end of the global health emergency on May 5, 2023, the confirmed cases of COVID-19 were estimated to be 765 million, and more than 6.9 million lives have been lost (http://www.who.int). At the same time, governments have trouble balancing between public health and economic crisis. Every level of the economy, from home and local to nationwide and global, has been seriously damaged due to the repetitive shutdown, which may lead to a global loss of $28 trillion by 2025, according to the International Monetary Fund (IMF) report [6, 7]. In addition, the emergencies, along with quarantine and social isolation, also spread psychological and emotional impacts, including stress, depression, anxiety, frustration, fear, and anger [8, 9]. Although the vaccine had been developed, approved, and inoculated with an unprecedented timeline, it took two more years until the end of the pandemic due to the repetitive waves and surge of the new variants [10, 11].

SARS-CoV-2 is the third emergence of highly pathogenic human coronaviruses (hCoV) during the last two decades, after SARS-CoV in 2003 and MERS-CoV in 2012 [12, 13], and there are commonalities in these three viruses. According to an analysis of genetic sequencing, SARS-CoV-2 is classified as betacoronavirus B lineage; its similarity to SARS-CoV and MERS-CoV was estimated to be ~ 79% and ~ 50%, respectively. Also, more than 96% of the SARS-Cov-2 genome matched with the bat coronavirus RaTG13, implying the zoonotic origin of these three viruses [14]. Unlike other coronaviruses, human coronaviruses originate from animal infections through a process called “spillover.” [15] This spillover means these viruses are almost new to the human immune system and thus result in highly dangerous outcomes through human-to-human transmission. However, the aftermath of the present SARS-CoV-2 pandemic is strikingly different from the previous SARS-CoV and MERS-CoV epidemics due to the ultra-fast, population-scale, and globe-wide transmissibility [16]. At the beginning of the pandemic, the average reproductive number was estimated to be 3.28, where one infected person would infect approximately three other people [14]. This number is much higher than SARS-CoV (~ 1.8) and MERS-CoV (< 1.0). In terms of the total infected cases, SARS-CoV-2 far surpasses SARS-CoV (8,096 cases) and MERS-CoV (2,553 cases). Although the fatality rate of SARS-CoV-2 is relatively low compared to those of the other two viruses, more contagious characteristics have caused the loss of millions of lives [17, 18].

One of the primary concerns of COVID-19 is asymptotic cases. Since infected individuals present with a wide range of symptoms, some act as silent spreaders, where these individuals are unaware of an infection. Numerous research indicate that asymptomatic individuals are as infectious as symptomatic individuals [19]. Therefore, the first step in containing the highly contagious virus is accurate viral detection at the early stage of the infection, followed by the proper measures for the infected people [20]. Early detection is a basic premise for all counteracting strategies, including quarantines, travel restrictions, contact tracing, and social distancing, for the COVID-19 pandemic because it enables us to find and isolate the infected individuals before they contact uninfected people [21].

Unfortunately, the reverse transcription-polymerase chain reaction (RT-PCR), which has served as the gold standard diagnostic method during the pandemic, is insufficient to fully address these exponentially increasing infection cases because it is neither time-efficient, cost-efficient, nor easily accessible. Even though rapid antigen tests (RATs) provide simple and efficient large-scale tests, their low sensitivity is not enough as an alternative to RT-PCR. In this context, many researchers have devoted themselves to developing alternative or complementary diagnostic tools for detecting SARS-CoV-2. Among these new techniques, nanobiosensors, which detect biological events at the nanoscale with the help of nanomaterials, have presented a new opportunity thanks to their conceptual design to reach ultrasensitive detection and quantification. Supposing that nanobiosensors provide accurate and reliable diagnostic results, they would be useful in lightening the burden of RT-PCR tests and increasing the possibility of early detection. In addition, nanobiosensors allow us to operate versatile anti-viral strategies for decision-making and rapid implementation against the emergence of infectious viral diseases.

## Conspectus of this review

**Fig. 1:**
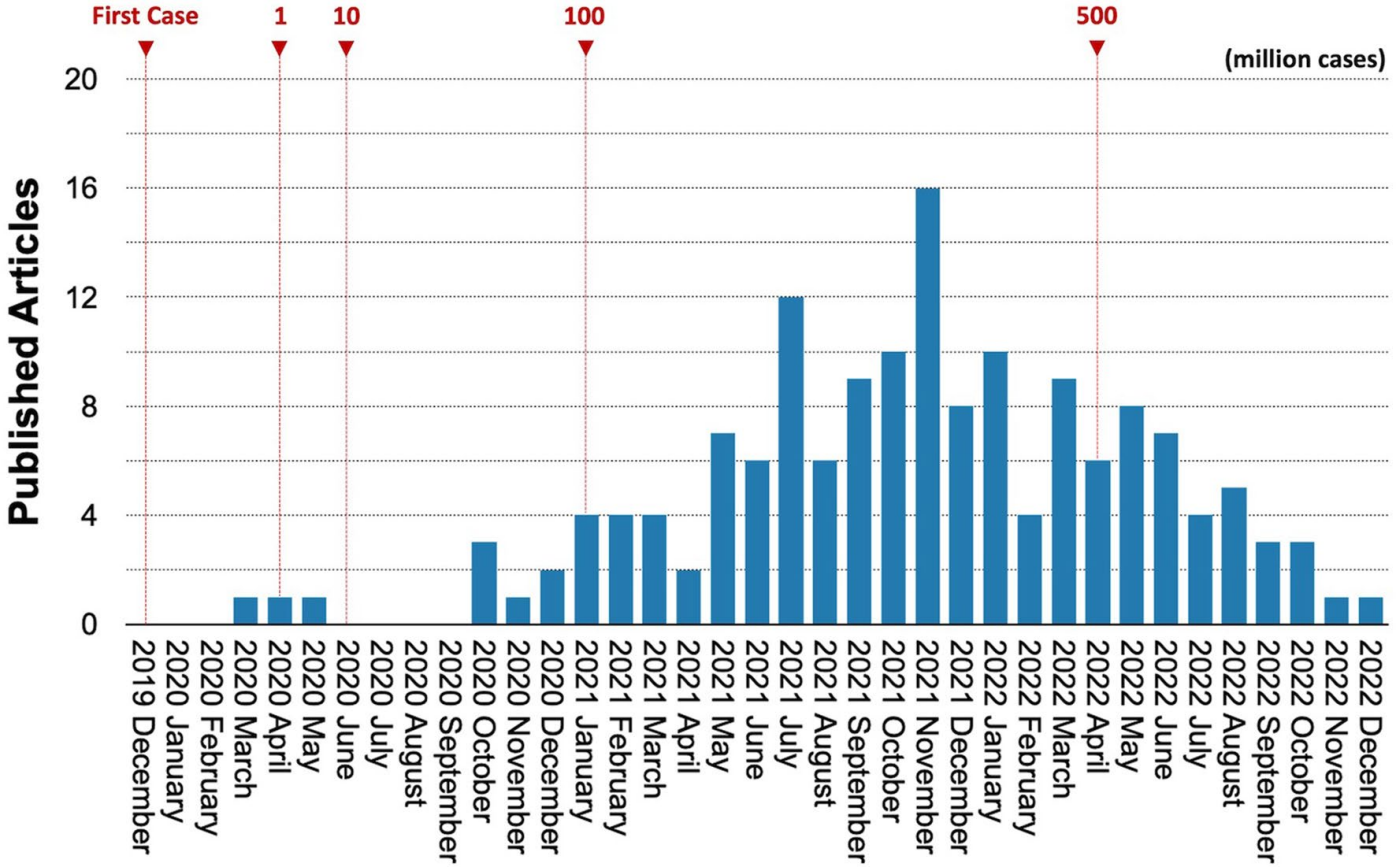
The distribution of articles focused on detecting SARS-CoV-2 antigens over time. A total of 158 published articles published between January 2020 and December 2022 (36 months) were collected for this review. The numbers above the graph represent the timeline of COVID-19 infection cases

This review is organized to focus on significant issues in the diagnosis of SARS-CoV-2 and the recent advances in nanobiosensors. The contents begin with a brief description of current diagnostic methods for SARS-CoV-2 (Sect. 3). Next, we summarize the recently reported nanobiosensors and their performance (Sect. 4) and then discuss the current challenges of nanobiosensors in the midst of the global pandemic in terms of the development of point-of-care (POC) diagnostics (Sect. 5). Lastly, we summarize the contents of this review focusing on the post-COVID era and potential future infectious diseases. The articles of interest were collected and extracted from PubMed, Scopus, Web of Science, and Google Scholar. Among the articles describing nanobiosensors for the detection of SARS-CoV-2, we particularly focused on antigen-detecting technologies as an alternative concept to current diagnostics like molecular tests and antibody testing kits. Also, we chose the articles published within three years after the SARS-CoV-2 outbreak (36 months). The total number of the included articles in this category is 158. The distribution of the articles of interest is presented in Fig. 1. The earliest publication date was March 27, 2020, and the latest publication date was December 30, 2022. We numbered the articles in chronological order in the group of the identical detection technique and marked them with an article ID consisting of one character and two-digit numbers (e.g., A00).

## Current diagnostic methods

The diagnosis of COVID-19 is not much different from that of other respiratory viral infections [22]. Currently, there are three major approaches to detecting SARS-CoV-2: (a) molecular tests, (b) antibody (serological) tests, and (c) antigen tests. These tests are based on diagnostic targets and their detection mechanisms. In the early outbreak, as a desperate measure, the infection was often confirmed using computed tomography (CT) based on abnormal features (e.g., varied opacities of lungs) [23]. One early study reported 85% of symptomatic and 50% of non-symptomatic COVID-19 patients had abnormal opacities in a CT scan ⁠[24, 25].⁠ However, as those data indicate, the CT-based diagnosis showed a clear limitation in distinguishing COVID-19 from other viral pneumonia. In addition, this expensive imaging method, which requires highly specialized equipment and trained personnel, makes it hard to contribute to the early detection of COVID-19.

### Molecular tests

Like other viral infections, RT-PCR has been considered the gold standard for SARS-CoV-2 identification [16]. This method detects the presence of viral RNAs (N, E, S, PRF1ab, ORF3a, and ORF7ab genes) in the sample, so identifying an active infection is possible, regardless of symptomatic or asymptomatic symptoms since this molecular test can determine the presence of RNA. Currently, RT-PCR has been deployed as a routine diagnostic process and is the most sensitive and reliable diagnostic tool today. According to the previous literature, RT-PCR can detect as low as nine copies of the virus per milliliter [26]. However, RT-PCR has some drawbacks, including a long turnaround time, high cost, labor-intensive protocol, and instrument-intensive processes with complex sample preparation under biosafety conditions. More importantly, RT-PCR is not free from false negatives due to the variance of the viral RNAs and the difficulties in nasopharyngeal swabbing. Numerous reports mention negative SARS-CoV-2 cases despite clinical symptoms of COVID-19 and suspicious CT images. There are disparities among the reports, but the average false-negative rate of RT-PCR was estimated to be 30–40% [27]. Also, the unstable nature of the RNA virus makes identification more difficult when the samples are not properly stored [28]. The low viral presence in the upper respiratory tract in both the early and late stages of the infection is problematic; therefore, individuals who were once infected and then recovered remain asymptomatic and would not be detected by RT-PCR test [19]. Generally, RT-PCR utilizes primers for different genes, which reflect the variation of viral RNA sequences. Thus, mutations in the primers and probes cause mismatches, thereby decreasing the performance of the assay.

### Antibody tests

The presence of immunoglobulin M (IgM) and immunoglobulin G (IgG) against SARS-CoV-2 is evidence of viral infection. Detecting IgM and IgG antibodies is an indirect approach that monitors the outcome of the dynamic humoral response of the infection, not the virus itself or parts thereof. However, this method is a more reasonable way to diagnose COVID-19 when considering the natural defense system of the human body [29]. Usually, as a primary immune response, neutralizing antibodies are found within 14 days after infection [30]. Unlike RT-PCR, serological tests, which identify antibodies in biological fluids, can be utilized to detect a past infection and the current level of immunity. For example, IgM is the first responder during the course of infection, and its amount rapidly increases during acute infection phases [31]. IgM can be detected after 3–5 days of the onset, and its level increases until it peaks around two weeks after the infection and then decreases to a near-background level. However, IgG can be an indicator of a current or prior infection. IgG reaches a detectable level after one week of infection and is maintained at a high concentration for a long time, even after seven weeks [32]. Since serological tests are not relevant to the presence of symptoms, these tests are effective for asymptomatic individuals as well [33]. Currently, there are several methods for serological binding assays, including enzyme-linked immunosorbent assays (ELISA), lateral flow immunoassays (LFIA), or Western blot-based assays [34, 35]. However, one critical drawback in serological tests is that the immune response usually takes a certain amount of time to produce antibodies after infection. This delay means that antibody tests depend on the produced antibody concentrations, and there is a gap between symptom onset and testing positive.

### Antigen tests

Unlike antibody tests, antigen tests directly detect parts of or the whole virus itself. Similar to other coronaviruses, SARS-CoV-2 consists of 29 proteins, including four structural proteins: S (spike), N (nucleocapsid), E (envelope), and M (membrane). Among these four proteins, S protein and N protein are accessible and can thus be considered candidates to detect SARS-CoV-2. One study even profiled the concentration range of S protein and N protein from patients [36].

S protein has especially been considered a rational target for nanobiosensors because of its form and function. A structural study using cryoelectron microscopy and tomography revealed that each virion has 24 × 9 perfusion S trimers, so it is possible to roughly estimate that each virus contains up to 100 S proteins [37]. S protein exists on the spikes protruding from the surface of virus particles, so detecting S protein closely correlates with detecting the virus itself. Furthermore, form follows function. Because S protein plays a role in entering the host cells, many researchers have pointed out that S protein is a critical target closely related to infectivity and pathogenesis [38]. However, S protein is limited as a target for antigen tests. First, there are different spatial orientations of S protein [39, 40]. Since the S protein has three orientations (RBD up, one RBD down, and two RBD down), the performance of the antigen test might depend on the position of the RBD. More importantly, considering that major mutations occur in S protein, a constant response against the newly emerging variants is hard to expect with S protein targeting.

In addition, detecting N protein is considered equivalent to detecting SARS-CoV-2 itself because N protein participates in the synthesis and translation of SARS-CoV-2 RNA [41]. Interestingly, there is evidence that the N protein has even higher immunogenicity than the RBD of the S protein [42]. Studies under identical conditions with purified S and N proteins usually show that N protein is an advantageous target. However, it is difficult to compare results from a model study to clinical samples. Some other research has pointed out that N protein targeting is less effective than S protein targeting using clinical samples [43]. This difference might be because of the position and functional role of the N protein, its location compared to the S protein, and its release only after host cell entry.

Like antibody tests, various types of immunoassays, such as ELISA and LFIA, can be utilized to detect S or N proteins. The commercial ELISA kits are provided by major companies like Abcam and Invitrogen. The sensitivity of these kits is in the range of around 10^–13^ to 10^–14^ M, in spite of the fact that the exact specification varies from batch to batch. Another limitation of ELISA is a narrow dynamic range with less than two orders. In the meantime, the commercial RAT kits were also supplied owing to their simple and rapid detection, enabling us to conduct at-home testing. However, commercial RAT kits have failed to be a counterpart of RT-PCR testing due to their low sensitivity. The possibility of infection cannot be ruled out by the negative results on the RAT kit, while even positive results should be verified once again using RT-PCR testing. It means that the possibility of early diagnosis using the RAT kits is quite low, and their testing results are not likely to contribute to the virus containment strategy.

## Nanobiosensors for the detection of SARS-CoV-2 antigen

Although most strategies and concepts for nanobiosensors have already been suggested during the past few decades, the urgent situation under the COVID-19 pandemic highlights their importance more than ever. Initially, nanobiosensors gained much attention due to the potential of nanomaterial-assisted enhancement of weak signals from biological events. Thanks to the high surface-area-to-volume ratio, nanomaterials can amplify signals of biological events, thus enabling us to detect a low concentration of analytes [44, 45]. Until now, however, nanobiosensors remained a laboratory practice because of the lack of accuracy, reliability, and validity. For example, fabrication errors among the samples are sometimes hard to control precisely due to uncontrollable variations [46]. Also, the sensitive characteristics of the nanomaterials are clear, while the mechanism of molecular recognition is ambiguous [47].

**Fig. 2:**
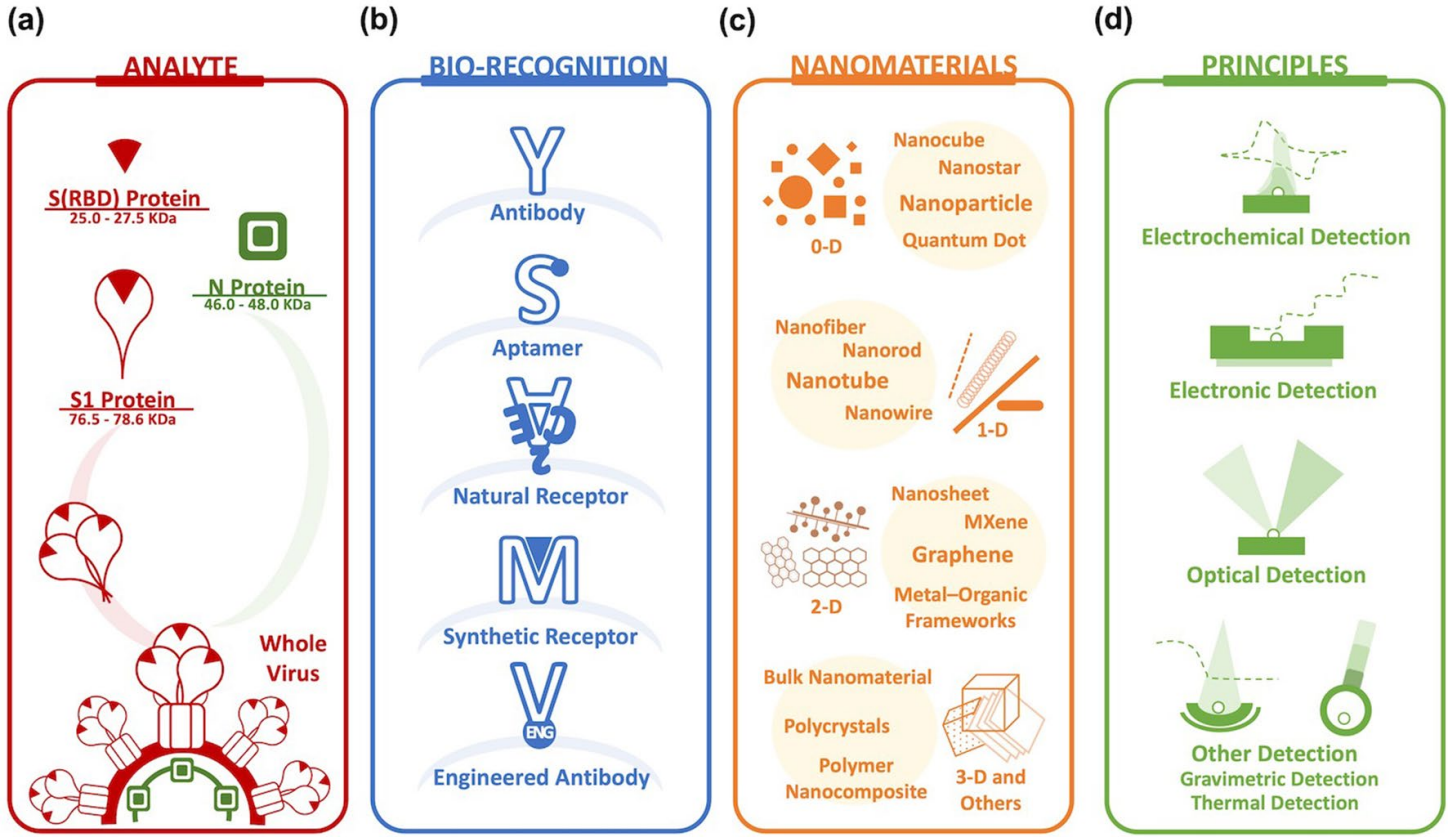
The design of nanobiosensors to detect SARS-CoV-2. The research is categorized by the following factors. **a** target (S1 protein, S(RBD) protein, and N protein); **b** biorecognition elements (antibody, aptamer, natural receptor, synthetic receptor, and engineered antibody); **c** materials and nanomaterials (0-D, 1-D, 2-D, 3-D, and others); **d** principles and transduction mechanism (electrochemical, electronic, optical, gravimetric, thermal, and others)

Previously, nanobiosensor studies have targeted various disease-related molecules, from glucose and cancer markers to potential carcinogens [48–50]. Viruses also have long been a target of interest, including influenza [51], human immunodeficiency virus (HIV) [52, 53], Ebola [54], Zika [55], dengue [56], measles [57], and norovirus [58]. When we narrow down the range to betacoronaviruses, nanobiosensors for the detection of SARS-CoV [59, 60] and MERS-CoV [61] were reported. However, the amount of SARS-CoV-2 research is unprecedented in many ways. The global scale of evolving threats of SARS-CoV-2 accelerated the research throughout all related fields, including nanobiosensors. This phenomenon reflects the urgent need for new tools and methods to detect the infectious virus. In this subsection, we classify the design of the reported nanobiosensors from 158 articles in terms of target analytes, biorecognition elements, nanomaterials, transduction mechanisms, experimental conditions, and evaluation criteria (Fig. 2).

### Target antigen

**Fig. 3:**
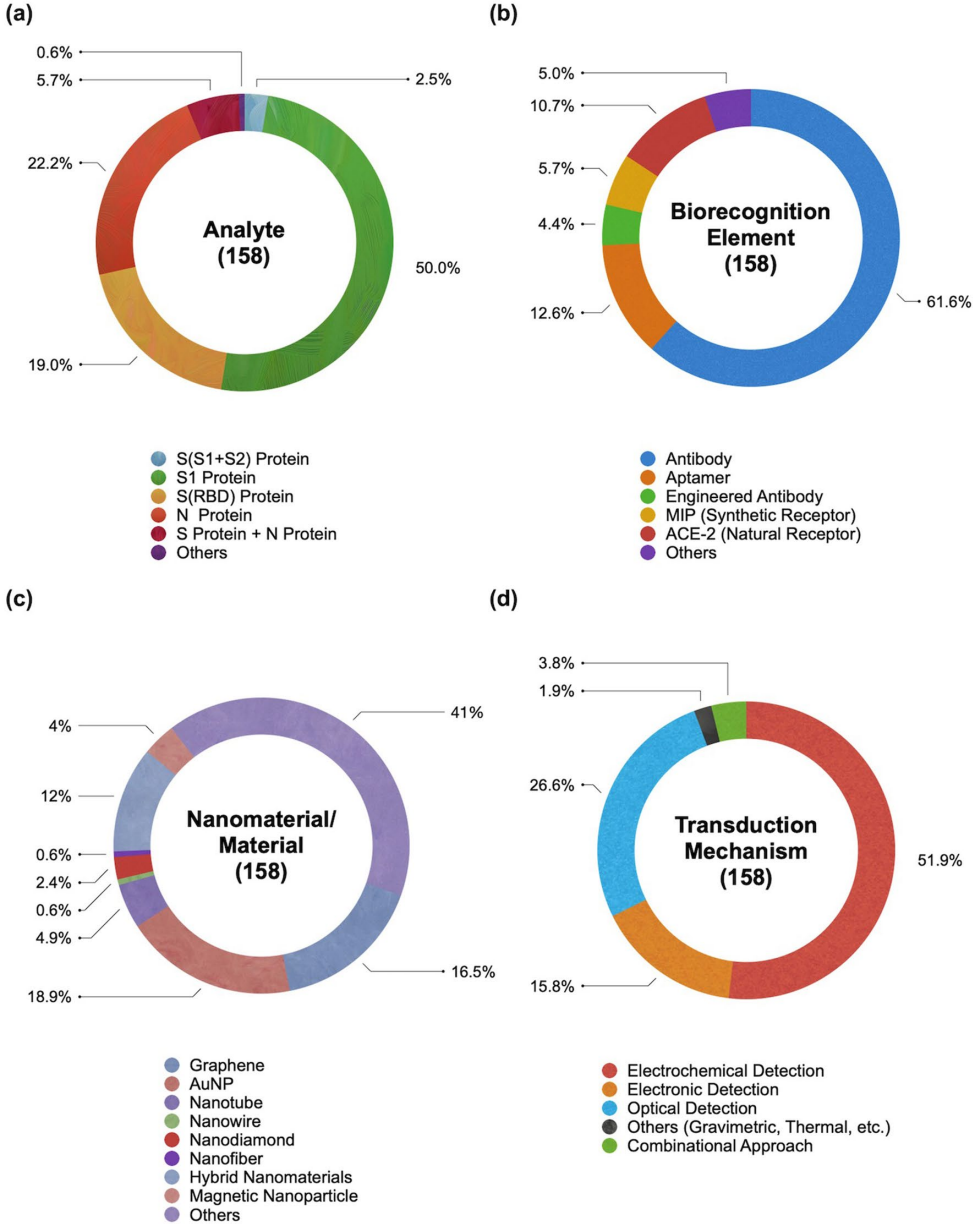
The distribution of nanobiosensors for the detection of SARS-CoV-2 antigen. **a** analyte; **b** biorecognition element; **c** nanomaterial and material; **d** transduction mechanism

The target antigens of nanobiosensors are mainly divided into two major forms (S protein and N protein) and four categories: S(S1 + S2) protein, S1 protein, S(RBD) protein, and N protein (Fig. 3a). These proteins are available on the market as recombinant proteins expressed from host cells. Their molecular weight is slightly different but somewhat consistent in specific ranges: S1 protein (76.5–78.6 kDa), S(RBD) protein (25.0–27.5 kDa), and N protein (46.0–48.0 kDa). Also, at least nine articles tested their scheme using both S and N proteins on a single platform. Two other protein targets were rarely reported: protease (N03) and ferritin (H14). Protease plays a role in viral replication, and hyperferritinemia is found in patients with poor clinical progress. Table 1 shows that more than half of the articles targeted the S1 protein (50.0%), followed by the N protein (22.2%) and S(RBD) protein (19.0%).

**Table 1:** The overall summary of articles of interest in this review

Transduction mechanism	ID	Detection methods	Total	Target analyte^b^						Biorecognition element^c^						Note
				S (S1 + S2)	S1	S (RBD)	N	S1 + N	Others	Antibody	Aptamer	Engineered antibody	ACE-2	MIP	Others	
Electrochemical detection (EC)	A	Differential Pulse Voltammetry (DPV)	19	1	8	4	5	1	–	13	2	–	2	2	–	–
	B	Squarewave voltammetry (SWV)	13	–	8	1	4	–	–	8	3	–	1	1	–	–
	C	Chronoamperometry (CA)	8	–	5	1	2	–	–	6	–	–	1	1	–	–
	D	Potentiometry (POT)	4	–	4	–	–	–	–	–	–	–	–	1	3	–
	E	Electrochemical Impedance Spectroscopy (EIS)	31	–	15	5	11	–	–	18	5	1	5	1	1	–
	F	Organic Electrochemical Transistor (OECT)	2	–	–	2	–	–	–	1	–	1	–	–	–	–
	G	Photoelectrochemistry (PEC)	5	2	1	1	1	–	–	2	2	1	–	–	–	–
Electronic detection (EL)	H	Field-effect Transistor (FET)	19	–	13	1	2	3	–	17	1	–	2	–	–	–
	I	Others	6	–	6	–	–	–	–	3	–	1	2	–	–	–
Optical detection (OP)	J	Fluorescence	7	–	1	1	3	2	–	4	2	–	–	–	1	–
	K	Chemiluminescence	2	–	–	1	1	–	–	2	–	–	–	–	–	–
	L	Surface Plasmon Resonance (SPR) spectroscopy	7	–	3	2	2	–	–	4	2	1	–	–	–	–
	M	Localized Surface Plasmon Resonance (LSPR)	3	–	1	1	1	–	–	1	–	–	1	1	–	–
	N	Fiber optics	6	1	3	0	1	–	1	3	1	–	–	1	1	–
	O	Surface-enhanced Raman scattering (SERS) spectroscopy	12	–	6	4	1	1	–	5	2	2	1	–	2	–
	P	Colorimetric	3	–	2	0	1	–	–	2	–	–	1	–	–	
	Q	Others	2	–	1	1	–	–	–	2	–	–	–	–	–	–
Mechanical/gravimetric/thermal detection (ME/GR/TH)	R	Microcantilever	1	–	–	–	–	1	–	1	–	–	–	–	–	–
	S	Magnetic Particle Spectroscopy (MPS)	1	–	–	–	–	1	–	1	–	–	–	–	–	–
	T	Thermal assay	1	–	–	1	–	–	–	–	–	–	–	1	–	–
Combinational approach (CA)	U	Multiple methods	6	–	2	4	–	–	–	5	–	–	1	–	–	–
SUM^a^			158	4	79	30	35	9	1	98	20	7	17	9	8	–

^a^Article collection period (3 year after outbreak; from January 2020 to December 2022; 36 Months)

^b^*S* Spike protein, *S1* S1 unit of S protein, *S2* S2 unit of S protein, *S(RBD)* receptor binding domain of S protein, *N* nucleocapsid protein

^c^*ACE-2* Angiotensin Converting Enzyme-2, *MIP* Molecularly Imprinted Polymer

### Biorecognition elements

The biorecognition element is a critical component of the nanobiosensor that recognizes the target analytes. There have been several bioreceptors, including antibodies, aptamers, and enzymes. In the SARS-CoV-2 studies, five kinds of biorecognition elements have been introduced: antibodies (anti-S antibody, anti-S1 antibody, anti-S2 antibody, anti-N antibody, anti-S(RBD) antibody), aptamers, engineered affinity proteins, natural receptors (ACE2 molecule), synthetic receptors (MIP), and others (Fig. 3b). Table 1 shows that immunosensors were used in over 61.6% of the total studies because of their excellent affinity, versatile applicability, and affordability of antibodies. Besides them, the portion of studies that used aptasensors, ACE2-based sensors, and MIP-based sensors are 12.6%, 10.7%, and 5.7%, respectively.

### Nanomaterials and other materials

The nanomaterials utilized varied from zero-dimensional (0D) to three-dimensional (3D) forms (Fig. 3c). Metallic nanoparticles (AuNPs, AgNPs, PtNPs, and PdNPs) were one of the most preferred nanomaterials because they are simple but strong signal amplifiers in every kind of detection mechanism, from plasmonic sensors to electrochemical sensors. In the meantime, the graphene family (graphene, graphene oxide, laser-scribed graphene, laser-engraved graphene) has also been preferred due to their extraordinary surface-to-volume ratio and conductivity. These two major nanomaterials occupy 18.9% and 16.5% of the study. Other nanomaterials utilized were other metallic nanomaterials (nanostars, nanorods, nanocubes, and nanowires), other carbon nanomaterials (nanotubes, nanofibers, carbon black, carbon dots, nanodiamond), magnetic nanoparticles, quantum dots, silica nanoparticle, hybrid nanocomposites, metal oxide frameworks (MOFs), 2-dimensional transition metal carbide and nitride (MXene), transition metal chalcogenides (TMCs). In the meantime, polymer, lipid, and hydrogel were also introduced as a matrix, carrier, or functional layer. Adopting polymeric material like a hydrogel is advantageous in concentrating viral analytes to elicit a greater signal [62, 63]. In fact, this has also been a materialization strategy for nanobiosensor to detect and enrich biomarkers [64–66].

### Transduction mechanism

The nanobiosensors are usually categorized under a transduction mechanism that converts the biorecognition event into a measurable signal. In this review, we classified the studies into four main categories: electrochemical (82/158), electronic (25/158), optical (42/158), and other (3/158) nanobiosensors (Fig. 3d). Each mechanism has its own characteristics and each detection method has pros and cons. (a) Electrochemical nanobiosensors occupied the greater part of the research because of their simplicity, affordability, portability, and user-friendliness [67]. Since electrochemical nanobiosensors require a relatively small volume, they can be implemented as a miniaturized device. On the other hand, electrochemical nanobiosensors are sensitive to the surrounding environment; signal interference from the redox reaction of background materials or ionic buffer can affect the performance. They are further divided into voltammetry, amperometry, potentiometry, electrochemical impedance spectroscopy (EIS), and others. (b) Electronic nanobiosensors, often represented by a field-effect transistor (FET), are also anticipated to be an ideal principle for ultrasensitive, label-free, and real-time detection with a minimal amount of sample. However, these transistor-based platforms have an optimization issue that causes inter-device and/or intra-device variation. Also, the possible ionic buffer interference implies the challenges in the operation using real sample. (c) Optical biosensors, one of the major operating principles, occupied almost a quarter of the research. They offer a safe and straightforward measurement of disease-related molecules [68]. In a broad sense, the basic concept of optical biosensors is very close to the sensitive, rapid, affordable, quantitative, and non-invasive or non-ionizing version of biomedical imaging. Still, they usually require relatively bulky devices compared to other categories of nanobiosensors, and some of them require additional signal probes or reporters. The representative optical biosensors are based on colorimetry, fluorescence, chemiluminescence (CL), surface plasmon resonance (SPR), localized surface plasmon resonance (LSPR), biolayer interferometry (BLI), and surface-enhanced Raman scattering (SERS). This category shows wide variation in principle, having benefits and drawbacks. For example, SPR enables label-free and real-time detection, whereas it does not discriminate the non-specific binding. SERS achieves single-molecule level sensitivity but often suffers from batch-to-batch reproducibility. (d) Other transduction mechanisms, gravimetric and thermal nanobiosensors, have rarely been reported in SARS-CoV-2 research. Gravimetric nanobiosensors are advantageous in simplicity, but their sensitivity is often unsatisfactory. Thermal nanobiosensors are free from optical and ionic disturbance, but the non-specific heating effect needs to be problematic. (e) In some cases, multiple transduction mechanisms were integrated into one platform.

### Experimental conditions

The basic experimental condition includes tests using target antigens diluted in a buffer at a certain concentration level. This condition optimizes the performance of the designed sensing system. For more realistic conditions, numerous studies spiked the antigens into the viral transport medium (VTM) or clinical samples obtained from healthy individuals. Although using a model virus is essential to assess the usability of the sensors, it is often not feasible due to safety issues. According to the CDC, the culture and passage of the virus should be conducted at biosafety level 3 (BSL-3), and routine diagnostic testing with inactivated virus samples also needs to be handled following biosafety level 2 (BSL-2) safety practices.

### Comparison of sensing performance

We evaluated the performance of the nanobiosensors based on the analytical sensitivity and detection range. The limit of detection (LoD) indicates the lowest concentration of analyte that can produce a statistically significant signal and should differ from a blank signal. For a fair comparison, we first transcribed the numbers precisely as the previous studies reported; however, the significant figures of the LoD were not suitable for comparison. Instead, we separately calculated the LoD in molar concentration for better understanding, indicated in parentheses with an asterisk (*). These estimated values are based on the information provided in each article, and the significant figures were unified with the same decimal places. In the meantime, the detection range of the sensors, usually referred to as linear range, dynamic range, or working range, was also collected for comparison. The plot displays the default format for performance evaluation, with the LoD (M) on the Y-axis and the detection range (order of magnitude) on the X-axis. As a result, the sensors show better performance when the marker is positioned close to the bottom-right corner of the graph. We also inserted a horizontal line (dotted) to roughly mark the physiological relevant level [69].

## Challenges for nanobiosensors

The nanobiosensors mentioned above promise rapid and ultrasensitive detection of SARS-CoV-2. Unfortunately, there are still several hurdles to applying nanobiosensors in real-world situations. In this subsection, we investigate the current diagnostic issues revealed during the COVID-19 global pandemic. Also, we discuss the implications of these advances in the post-COVID era. To suggest a standard for an impartial evaluation, we used ASSURED, one of the most widely used criteria for POC diagnostics. Although nanobiosensors do not need to be a form of POC testing, their POC conditions are also the utmost goal of nanobiosensors, especially in the detection of infectious diseases.

### ASSURED criteria

The ASSURED criteria suggested by the WHO is a set of requirements for developing practical diagnostics, especially in POC testing. In general, most current standard methods (e.g., RT-PCR, ELISA) have weaknesses in the ASSURED criteria, regarding affordable (A), sensitive (S), specific (S), user-friendly (U), rapid and robust (R), equipment-free (E), and deliverable to the end-user (D) standards.

#### Affordable tests (A)

Affordability indicates the cost-effectiveness of a diagnostic test (i.e., cost per test) while also considering a total socioeconomic burden. The importance of the socioeconomic area has greatly increased, especially with infectious diseases like COVID-19. RT-PCR, a staple of the current preventative strategy, is expensive. The final test cost is largely varied across countries. Usually, the cost of a single RT-PCR kit is between 100 and 125 USD [70, 71], and technologist costs are added separately. We should consider that this cost is not a one-off. The number of tests increases exponentially with the spreading of the virus. Every time an individual comes in contact with someone who is infected, they are required to get tests. Once an individual becomes infected, another test will be required after a certain period of quarantine (i.e., 7–14 days). In March 2020, the WHO estimated that around 10 to 30 tests were required for one positive case. In terms of both cost and labor, RT-PCR tests are quite a pressure on healthcare systems. Furthermore, we should also consider the set-up cost for a diagnostic laboratory. It is estimated that the average set-up cost for new diagnostic laboratories is around 15,000 USD per lab [72], which does not include labor and training costs.

In this context, the cost of the rapid antigen kits (5 to 10 USD per test), which also underlies the expanded test capacity, provides a practical guideline for nanobiosensors. Among the articles, 81/158 (51.3%) claimed to use a low-cost test. Although the low-cost claim implies substantial cost reduction, it does not always guarantee the eventual cost of a single test. There are a few ways to present the cost reduction of the invented methods. The first way is to focus on the inexpensive elements of the invention; for example, mass-producible electrodes, including glassy carbon (A06, B06, E06), screen-printed electrodes (A06, A08, C03, C06, E20), graphite leads (B07), ITO substrate (E07), and LSPR substrate (M03). Some platforms can be fabricated with cost-efficient methods, like electrochemical anodization (C01), microfluidic chip (E11), micro-electrode (E24), microcantilever (Q01), 3D printing (J05), or other relatively simple processes (B09, I01, I03, J03). The fundamental advantages of each transduction method, especially in electrochemical detection, can also be considered, though the description of each method often did not include the price in detail (B01, B05, B09, C07, E01, E08, E13, E14, K02). Interestingly, one study (H10) focused on the economical sampling method, like pool testing, because their sensor showed a high specificity even with the 1-in-10 pooled samples [73].

The cost of biorecognition receptors is hard to control and accounts for most of the overall cost. Antibodies are an expensive element of immunosensors. The price range of antibodies (100 μL) against S protein, S(RBD) protein, or N protein is usually between $400 and $600, depending on the providers, countries, and currencies. For these reasons, some researchers produced in-house antibodies in a more economical way (A07 and H19). Similarly, aptamers are more inexpensive than antibodies, so aptasensor research tended to emphasize that aptamers are relatively more affordable than antibodies (E09, G01). Deng et al. analyzed the cost of aptamer production and suggested that their assay required 1 pM. of aptamer per test, equivalent to $0.006 [38]. It is much inexpensive than commercial antibody ($0.01 to $0.04 per pM). The same logic applied to the MIP-based research because it is an economical and even robust alternative to antibodies and aptamers (A08, B10, E10, E25, M03, N01).

Similarly, some low-cost claims were based on the instrumentation costs (A15, C07, E17, E23, G02, H08). A few studies indicated commercial-but-inexpensive equipment, whereas others described their own customization at the lab scale. Using commercial chips may be an advantage of cost reduction. Qi et al. (E15) utilized a commercial MEA chip for their capacitive aptasensor [74]. Another interesting study (B04) utilized the children’s toy, Shrinky-Dink^©^ electrodes [75]. On rare occasions, several studies tried to present the estimated eventual cost of a single test with specific sums. For example, Torres et al. (E05) estimated the cost of their RAPID 1.0 biosensor to be $4.67, consisting of fabrication and functionalization costs [76]. Likewise, Salahandish et al. (E23) estimated the cost of their BiSense biosensor to be $40, including the customized Potentiostat [77].

#### Sensitive tests (S)

The early identification of SARS-CoV-2 infection is the most important issue in this subject. To do so, the sensors should be sensitive to detect a very small amount of virus or parts of the virus before the symptom onset. Some early reports indicate that viral loads of asymptomatic and symptomatic patients are not much different; therefore, theoretically, the identification of individuals with no or mild symptoms is possible [78]. The question is: how much should they be sensitive? It is axiomatic that the more sensitive the test, the better. Unlike traditional target analytes of nanobiosensors, there is no specific background level of viruses in humans, so the requirement for optimal sensitivity is somewhat complex and ambiguous. Furthermore, in this subject, the sensor should cover a wide range of analyte concentrations since the viral load and symptom onset timing of each individual is different. This implies the need for accurate quantification, overcoming simple Yes/No outcomes. For this reason, previous studies suggested various ways to discuss sensitive detection of SARS-CoV-2.

**Fig. 4:**
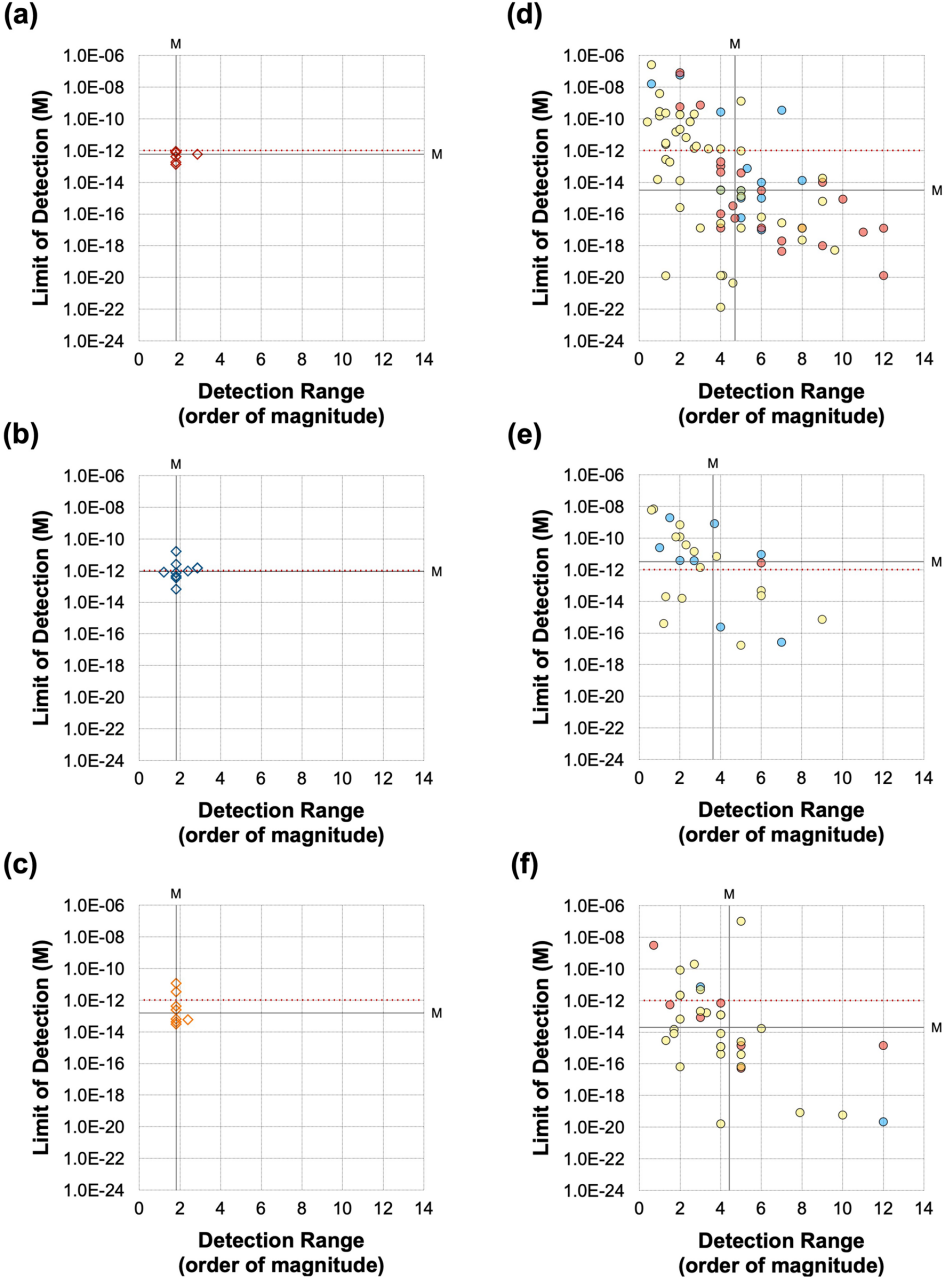
The performance of (**a**–**c**) commercial ELISA kits and (**d**–**f**) recent nanobiosensors for the detection of SARS-CoV-2 antigens. The X-axis and Y-axis display the limit of detection (M) and working range (order of magnitude): **a/d** S1 protein; **b/e** S(RBD) protein; **c/f** N protein. In the plot of nanobiosensors, orange, yellow and blue circles indicate electrochemical, electronic, and optical detection. The black solid lines indicate the median value of the group, and the red dotted line marks a picomolar (physiologically relevant) level of viral proteins

In terms of analytical sensitivity, which is an indicator of core performance using model samples with recombinant proteins, the nanobiosensors referred to in this review showed a largely varied LoD from the zeptomolar level to the nanomolar level (Table 2). There are several ways to set a reference point. One guideline can be set based on the mathematical modeling. According to the research, physiologically relevant levels of SARS-CoV-2 are around 7 × 10^6^ virions per milliliter [79]. Because this level is equivalent to approximately 10 fM, Stanborough et al. estimated ~ 0.25 pM of S protein as a physiologically relevant level [69]. Therefore, they concluded that sub-picomolar detection is required for antigen detection. In the same manner, we can assume that the physiologically relevant N protein level would also be around the picomolar level. Although the exact amount of N protein per virus is difficult to quantify, a recent analysis concluded that there is three times more N protein than S protein monomer in a single virus [80]. Another guideline for setting a reference point uses the sensitivity of commercial ELISA kits. As seen in Fig. 4, the performance of the ELISA is converged in a similar range, though there is a slight variation according to the provider and production batch. The LoD of ELISA spans around the picomolar level, where the physiologically relevant range is, and the detection range is usually one or two orders of magnitude. Therefore, the best performance of ELISA might distinguish the patients at the boundary, but it is still not satisfactory to detect the virus at the very early infection stage; thus, the advisable sensitivity of nanobiosensors should be better than this range. Figure 4 indicates that three-quarters of the nanobiosensor studies show a better LoD and detection range than the ELISA.

**Table 2:** The sensing performance of the representative SARS-CoV-2 nanobiosensors

ID	Design					Performance evaluation (protein)				Performance evaluation (model virus)					Note	
	Transduction mechanism	Detection technique	Materials/nanomaterials	Biorecognition element	Target antigen	Sample matrix^a^	Limit of detection (M)	Detection range (M)	Order	Model	Sample matrix^b^	Limit of detection	Detection range	Order	Publish date	References
A01	EC	DPV	Graphene oxide (GO)/Au nanostar	Antibody	S1	PBS	2.20 × 10^–21^	–	–	–					OCT 2020	[82]
A02	EC	DPV	Carbon black	Antibody	S1	PBS	1.83 × 10^–10^ (14 ng/mL)*	1.3 × 10^–10^ to 1.3 × 10^–8^	2	Inactivated virus	PBS	6.5 (PFU/mL)	Not determined	–	OCT 2020	[83]
					S1	Saliva	2.48 × 10^–10^ (19 ng/mL)*	1.3 × 10^–10^ to 1.3 × 10^–8^	2							
					N	PBS	8.39 × 10^–11^ (4 ng/mL)*	2.1 × 10^–10^ to 2.1 × 10^–8^	2	Inactivated virus	PBS	6.5 × 10^3^ (PFU/mL)	6.5 × 10^3^ to 6.5 × 10^5^ (PFU/mL)	2		
					N	Saliva	1.68 × 10^–10^ (8 ng/mL)*	2.1 × 10^–10^ to 2.1 × 10^–8^	2							
A03	EC	DPV	–	MIP	N	Lysis Buffer	1.50 × 10^–14^	2.0 × 10^–15^ to 1.1 × 10^–13^	1	–					JAN 2021	[84]
A04	EC	DPV	Metal–organic frameworks (MOF)/Au@Pt nanoparticles	Aptamer	N	N/A	1.74 × 10^–13^ (8.33 pg/mL)*	5.2 × 10^–13^ to 1.0 × 10^–9^	3	–					MAY 2021	[85]
A05	EC	DPV	Laser-scribed graphene	Aptamer	S(RBD)	0.1 M PBS	1.16 × 10^–10^ (2.9 ng/mL)*	2.0 × 10^–10^ to 2.0 × 10^–8^	2	–					JUN 2021	[86]
A06	EC	DPV	Graphene oxide (GO)	Antibody	S1	N/A	1.31 × 10^–20^ (1 ag/mL)*	1.0 × 10^–20^ to 1.3 × 10^–16^	4	–					JUL 2021	[87]
A07	EC	DPV	Au nanoparticles (AuNPs)	Antibody	S1	PB	6.30 × 10^–16^	1.0 × 10^–15^ to 1.0 × 10^–6^	9	–					OCT 2021	[88]
					S1	Saliva	1.20 × 10^–13^	Not determined	–							
A08	EC	DPV	Au/graphene	MIP	N	0.1 M KCl	3.00 × 10^–15^	1.0 × 10^–14^ to 2.0 × 10^–13^	1	–					NOV 2021	[89]
A09	EC	DPV	Au nanoparticles (AuNPs)	ACE-2	S1	Diluted saliva	4.45 × 10^–21^ (0.35 ag/mL)*	1.2 × 10^–19^ to 4.7 × 10^–15^	4	–					NOV 2021	[90]
A10	EC	DPV	Au nanoparticles (AuNPs)	Antibody	S1	PBS	1.31 × 10^–17^ (1 fg/mL)*	1.3 × 10^–16^ to 1.3 × 10^–11^	5	–					NOV 2021	[91]
A11	EC	DPV	g-C3N4/Au/WO3	Antibody	N	0.1 M PBS	6.52 × 10^–17^ (3 fg/mL)*	2.2 × 10^–16^ to 2.2 × 10^–14^	2	–					NOV 2021	[92]
A12	EC	DPV	Pd-Au nanosheet	Antibody	S1	PBS	9.50 × 10^–13^ (0.0072 ng/mL)*	1.0 × 10^–8^ to 1.0 × 10^–3^	5	–					DEC 2021	[93]
A13	EC	DPV	Graphene oxide (GO)/Au nanoparticles (AuNPs)	Antibody	N	PBS	8.31 × 10^–20^ (3.99 ag/mL)*	2.0 × 10^–20^ to 2.0 × 10^–12^	7	–					MAY 2022	[94]
A14	EC	DPV	Au nanorod	Antibody	S(RBD)	N/A	7.30 × 10^–16^	1.0 × 10^–15^ to 1.0 × 10^–6^	9	–					MAY 2022	[95]
A15	EC	DPV	Au nanoparticles (AuNPs)	Antibody	S1	Tris/ VTM	1.53 × 10^–10^	6.6 × 10^–9^ to 6.6 × 10^–1^	1	–					JUN 2022	[96]
A16	EC	DPV	Laser-scribed graphene (LSG)	ACE-2	S1	0.1 M PBS	6.54 × 10^–11^ (5.14 ng/mL)*	1.3 × 10^–11^ to 4.5 × 10^–9^	2	–					JAN 2022	[97]
					S2	0.1 M PBS	3.75 × 10^–11^ (2.09 ng/mL)*	1.8 × 10^–11^ to 3.6 × 10^–9^	2							
A17	EC	DPV	Au nanoparticles (AuNPs)	Aptamer	S1	PBS	1.40 × 10^–12^ (0.11 ng/mL)*	2.5 × 10^–11^ to 1.3 × 10^–8^	2	–					SEP 2022	[98]
A18	EC	DPV	Single-walled carbon nanotube (SWCNT)	Aptamer	S(RBD)	PBS	7.00 × 10^–9^	2.0 × 10^–8^ to 1.0 × 10^–7^	–	–					APR 2022	[99]
A19	EC	DPV	Ag/reduced graphene oxide	Antibody	S(RBD)	0.1 M PBS	2.62 × 10^–16^ (7.2 fg/mL)*	5.4 × 10^–13^ to 5.8 × 10^–9^	4	–					NOV 2022	[100]
B01	EC	SWV	Carbon nanofiber	Antibody	N	PBS	1.70 × 10^–14^ (0.8 pg/mL)*	2.1 × 10^–14^ to 2.1 × 10^–8^	6	–					DEC 2020	[101]
B02	EC	SWV	Graphene	Antibody	S1	PBS	2.60 × 10^–7^	2.7 × 10^–7^ to 1.0 × 10^–6^	–	Inactivated virus	Lysis buffer/PBS	5.5 × 10^5^ (PFU/mL)	Not determined	–	JAN 2021	[102]
B03	EC	SWV	Wrinkled gold	Aptamer	S1	10% Saliva	1.31 × 10^–17^ (1 ag/mL)*	1.3 × 10^–18^ to 1.3 × 10^–14^	4	–					FEB 2021	[75]
B04	EC	SWV	Au nanoparticles (AuNPs)	Antibody	N	PBS	8.33 × 10^–15^ (0.4 pg/mL)*	2.1 × 10^–14^ to 2.1 × 10^–9^	5	–					MAY 2021	[103]
B05	EC	SWV	Au cluster	Antibody	S1	0.1 M PBS	1.31 × 10^–22^ (0.01 ag/mL)*	1.3 × 10^–21^ to 1.3 × 10^–17^	4	–					MAY 2021	[104]
B06	EC	SWV	Au nanoparticles (AuNPs)	ACE-2	S1	0.1 M PBS	2.99 × 10^–15^ (229 fg/mL)*	1.3 × 10^–16^ to 1.3 × 10^–11^	5	Inactivated virus	VTM	2.07 (PFU/mL)	1.0 × 10^2^ to 1.0 × 10^5^ (PFU/mL)	3	JUL 2021	[105]
B07	EC	SWV	–	Aptamer	S1	PBS	1.49 × 10^–11^	Not determined	–	–					JUL 2021	[106]
B08	EC	SWV	Au microcuboid	Antibody	S1	10 mM PBS	2.76 × 10^–13^	5.0 × 10^–12^ to 1.0 × 10^–10^	4	–					AUG 2021	[107]
B09	EC	SWV	–	MIP	S1	PBS	1.50 × 10^–14^	2.7 × 10^–14^ to 1.9 × 10^–13^	1	–					NOV 2021	[108]
						NS	6.40 × 10^–14^	5.0 × 10^–14^ to 4.0 × 10^–13^	1							
B10	EC	SWV	Laser-engraved graphene	Aptamer	S(RBD)	0.1 M PBS	1.44 × 10^–11^ (0.36 ng/mL)*	2.0 × 10^–11^ to 1.0 × 10^–8^	2	–					JUL 2022	[109]
B11	EC	SWV	Graphene oxide (GO)	Antibody	N	0.01 M PBS	1.61 × 10^–20^ (0.76 ag/mL)*	2.1 × 10^–20^ to 2.1 × 10^–16^	4	–					SEP 2022	[110]
					N (Ο)		5.10 × 10^–21^ (0.24 ag/mL)*	2.1 × 10^–20^ to 2.1 × 10^–15^	5							
B12	EC	SWV	Au nanoparticles (AuNPs)	Antibody	N	10 mM PBS	5.65 × 10^–14^ (2.6 pg/mL)*	2.2 × 10^–13^ to 2.2 × 10^–8^	5	–					OCT 2022	[111]
B13	EC	SWV	Single-walled carbon nanotube (SWCNT)	Antibody	S1	PBS	2.00 × 10^–10^	1.0 × 10^–9^ to 5.0 × 10^–7^	2	Pseudovirus	DMEM + 10%FBS	10^6^ (copies/mL)	5.0 × 10^6^ to 1.0 × 10^7^ (copies/mL)	–	DEC 2022	[112]
						Filtered saliva	5.00 × 10^–10^	5.0 × 10^–9^ to 1.0 × 10^–7^	1							
C01	EC	CA	Cobalt-functionalized TiO2 nanotubes (Co-TNTs)	Antibody	S(RBD)	Buffer	7.00 × 10^–10^	1.4 × 10^–8^ to 1.4 × 10^–6^	2	–					OCT 2020	[113]
C02	EC	CA	–	Antibody	S1	N/A	1.27 × 10^–14^ (1 pg/mL)*	Not determined	–	Pseudotyped virus	N/A	4 × 10^3^ (copies/mL)	4.0 × 10^4^ to 4.0 × 10^7^ (copies/mL)	3	JAN 2021	[114]
C03	EC	CA	–	Antibody	S1	N/A	1.91 × 10^–12^ (150 pg/mL)*	1.9 × 10^–12^ to 1.3 × 10^–9^	2	Inactivated virus	N/A	29 (PFU/mL)	2.9 × 10^1^ to 2.9 × 10^2^ (PFU/mL)	1	MAY 2021	[115]
C04	EC	CA	Magnetic nanobeads	Antibody	N	Whole serum	1.04 × 10^–12^ (50 pg/mL)*	2.1 × 10^–13^ to 2.1 × 10^–10^	3	–					JUL 2021	[116]
					N	5X diluted serum	2.08 × 10^–13^ (10 pg/mL)*	2.1 × 10^–13^ to 2.1 × 10^–10^	3							
C05	EC	CA	–	MIP	S1	PBS	6.36 × 10^–11^ (5 ng/mL)*	1.3 × 10^–7^ to 3.2 × 10^–7^	–	–					NOV 2021	[117]
C06	EC	CA	–	Antibody	N	N/A	N/A	Not determined	–	Inactivated virus	VTMT	50 (PFU/mL)	2.2 × 10^2^ to 2.2 × 10^4^ (PFU/mL)	2	MAR 2022	[118]
C07	EC	CA	Magnetic nanobeads	ACE-2	S1	PBS	2.86 × 10^–10^ (22.5 ng/mL)*	1.3 × 10^–8^ to 1.3 × 10^–7^	1	Inactivated virus	PBS	1.2 × 10^–1^ (copies/mL)	1.0 × 10^0^ to 1.0 × 10^6^ (copies/mL)	6	MAR 2022	[119]
C08	EC	CA	–	Antibody	S1	Buffer	2.45 × 10^–12^ (0.19 ng/mL)*	6.4 × 10^–12^ to 1.3 × 10^–10^	1	–					MAR 2022	[120]
						Artificial Saliva	1.67 × 10^–12^ (0.13 ng/mL)*	6.4 × 10^–12^ to 3.9 × 10^–11^	–							
D01	EC	POT	–	Cell	S1	N/A	1.27 × 10^–17^ (1 fg/mL)*	1.3 × 10^–16^ to 1.3 × 10^–8^	8	–					MAY 2020	[121]
D02	EC	POT	–	Cell	S1	N/A	2.54 × 10^–17^ (2 fg/mL)*	2.5 × 10^–17^ to 2.5 × 10^–13^	4	–					JUN 2021	[122]
D03	EC	POT	–	Cell	S1	PBS	2.54 × 10^–16^ (20 fg/mL)*	2.5 × 10^–16^ to 2.5 × 10^–14^	2	–					DEC 2021	[123]
D04	EC	POT	–	MIP	S1	Saliva	1.27 × 10^–12^ (100 pg/mL)*	Not determined	–	H1N1, H3N2 virus	Saliva	200 (PFU/mL)	2.0 × 10^2^ to 1.0 × 10^3^ (copies/mL)	–	APR 2022	[124]
E01	EC	EIS	CuO_2_ nanocube	Antibody	S1	PB	5.23 × 10^–19^ (0.04 fg/mL)*	3.3 × 10^–18^ to 1.3 × 10^–8^	9	–					MAR 2021	[125]
E02	EC	EIS	–	ACE-2	S1	PBS	2.19 × 10^–11^ (1.68 ng/mL)*	1.3 × 10^–11^ to 1.3 × 10^–9^	2	Inactivated virus	VTM	38.6 (copies/mL)	1.0 × 10^3^ to 1.0 × 10^5^ (copies/mL)	2	MAR 2021	[126]
E03	EC	EIS	Pd nanoparticle	ACE-2	S1	PBS	1.31 × 10^–9^ (0.1 μg/mL)*	1.0 × 10^–9^ to 1.0 × 10^–4^	5	–					APR 2021	[127]
E04	EC	EIS	Boron-doped diamond (BDD)	Antibody	S1	PBS	1.31 × 10^–17^ (1 fg/mL)*	1.3 × 10^–17^ to 1.3 × 10^–14^	3	–					JUL 2021	[128]
E05	EC	EIS	–	ACE-2	S1	PBS	2.77 × 10^–17^ (2.18 fg/mL)*	1.3 × 10^–16^ to 1.3 × 10^–9^	7	Inactivated virus	VTM	1.16 (PFU/mL)	1.0 × 10^1^—1.0 × 10^6^ (PFU/mL)	5	JUL 2021	[76]
						VTM	8.00 × 10^–17^ (6.29 fg/mL)*	1.3 × 10^–16^ to 1.3 × 10^–11^	5							
						Saliva	1.77 × 10^–14^ (1.39 pg/mL)*	1.3 × 10^–15^ to 1.3 × 10^–9^	6							
E06	EC	EIS	Graphene oxide (GO)	Antibody	S(RBD)	PBS	5.45 × 10^–9^ (150 ng/mL)*	1.6 × 10^–8^ to 6.4 × 10^–8^	–	–					JUL 2021	[129]
E07	EC	EIS	Conducting nanocomposite	Engineered antibody	S(RBD)	N/A	1.66 × 10^–17^ (0.58 fg/mL)*	3.4 × 10^–17^ to 3.4 × 10^–12^	5	–					JUL 2021	[130]
E08	EC	EIS	Au nanoparticles (AuNPs)	Aptamer	S1	PBS (+ Salt)	1.3 × 10^–12^ (66 pg/mL)*	1.0 × 10^–11^ to 2.5 × 10^–8^	3	–					AUG 2021	[131]
E09	EC	EIS	Carbon nanodiamond	Aptamer	N	Diluted Serum	3.9 × 10^–16^	1.0 × 10^–15^ to 1.0 × 10^–10^	5	–					OCT 2021	[132]
E10	EC	EIS	–	MIP	S(RBD)	N/A	2.0 × 10^–14^ (0.7 pg/mL)*	5.7 × 10^–14^ to 1.1 × 10^–12^	1	–					OCT 2021	[7]
E11	EC	EIS	ZnO nanoparticle/graphene	Antibody	N	PBS	6.74 × 10^–14^ (3.1 pg/mL)*	2.2 × 10^–13^ to 2.2 × 10^–11^	2	–					NOV 2021	[133]
E12	EC	EIS	–	ACE-2	S1	N/A	3.94 × 10^–9^ (299.3 ng/mL)*	9.2 × 10^–9^ to 2.0 × 10^–8^	1	–					NOV 2021	[134]
				CD-147 Receptor	S1	N/A	5.1 × 10^–10^ (38.99 ng/mL)*	6.6 × 10^–9^ to 6.6 × 10^–8^	1							
E13	EC	EIS	–	Antibody	S1	N/A	2.3 × 10^–18^ (0.179 fg/mL)*	1.3 × 10^–24^ to 1.3 × 10^–16^	8	Inactivated virus	PBS	7.0 × 10^–1^ (PFU/mL)	N/A	N/A	NOV 2021	[135]
E14	EC	EIS	Modified MWCNT/graphene	Antibody	S1	PBS	1.3 × 10^–20^ (0.001 fg/mL)*	1.5 × 10^–20^ to 3.2 × 10^–19^	1	–					NOV 2021	[136]
E15	EC	EIS	–	Aptamer	N		6.6 × 10^–17^ (3.16 fg/mL)*	2.1 × 10^–16^ to 2.1 × 10^–11^	5	–					DEC 2021	[74]
E16	EC	EIS	SiO_2_@UiO-66 nanocomposite	ACE-2	S1		1.3 × 10^–15^ (100 fg/mL)*	1.0 × 10^–10^ to 1.0 × 10^–5^	5	–					JAN 2022	[137]
E17	EC	EIS	Carbon/graphene@PEDOT:PSS	Antibody	N	PBS	2.52 × 10^–15^ (116 fg/mL)*	2.2 × 10^–15^ to 2.2 × 10^–10^	5	–					JAN 2022	[77]
							3.26 × 10^–15^ (150 fg/mL)*									
E18	EC	EIS	Au nanoparticles (AuNPs)	Antibody	N	PBS	1.0 × 10^–7^ (0.48 fg/mL)*	3.3 × 10^–17^ to 3.3 × 10^–12^	5	–					JAN 2022	[138]
E19	EC	EIS	Zinc oxide/reduced graphene oxide (bbZnO/rGO)	Antibody	N	PBS	4.1 × 10^–16^ (21 fg/mL)*	2.2 × 10^–14^ to 2.2 × 10^–10^	4	–					FEB 2022	[139]
E20	EC	EIS	Au Nanoparticles (AuNPs)	Antibody	S1	PBS	3.16 × 10^–15^	1.0 × 10^–11^ to 1.0 × 10^–7^	4	Inactivated virus	N/A	1.0 × 10^6^ (PFU/mL)	Not determined	–	FEB 2022	[140]
E21	EC	EIS	Magnetic nanoparticle	Antibody	S1	PBS	1.18 × 10^–11^ (0.93 ng/mL)*	3.2 × 10^–11^ to 2.5 × 10^–9^	1	–					MAR 2022	[141]
						PBS	6.74 × 10^–12^ (0.53 ng/mL)*	1.3 × 10^–11^ to 2.5 × 10^–9^	2							
				Antibody	S2	PBS	1.78 × 10^–11^ (0.99 ng/mL)*	1.8 × 10^–11^ to 2.6 × 10^–9^	2							
						PBS	1.33 × 10^–11^ (0.75 ng/mL)*	4.5 × 10^–11^ to 1.8 × 10^–9^	1							
E22	EC	EIS	Gold nanostar	Antibody	N	PBS	1.25 × 10^–13^ (6 pg/mL)*	2.1 × 10^–13^ to 2.1 × 10^–9^	4	–					APR 2022	[142]
						Diluted Saliva	1.25 × 10^–13^ (6 pg/mL)*	2.1 × 10^–13^ to 2.1 × 10^–9^	4							
E23	EC	EIS	Carbon PEDOT:PSS graphene	Antibody	N	PBS	1.22 × 10^–15^ (56 fg/mL)*	2.2 × 10^–14^ to 2.2 × 10^–10^	4	–					JUN 2022	[143]
							1.48 × 10^–15^ (68 fg/mL)*									
E24	EC	EIS	–	Antibody	N	PBS	2.17 × 10^–12^ (0.1 ng/mL)*	2.2 × 10^–12^ to 2.2 × 10^–10^	2	–					JUN 2022	[144]
E25	EC	EIS	–	Aptamer	S(RBD)	0.1 M PBS	7.00 × 10^–12^	1.0 × 10^–11^ to 6.4 × 10^–8^	3	–					JUN 2022	[145]
E26	EC	EIS	Carbon nanodiamond	Antibody	S1	10 mM PBS	1.89 × 10^–13^	2.5 × 10^–13^ to 8.0 × 10^–12^	1	–					MAY 2022	[146]
E27	EC	EIS	–	Synthetic Peptide	S1	N/A	2.32 × 10^–10^ (18.2 ng/mL)*	6.4 × 10^–10^ to 1.3 × 10^–10^	1	–					MAY 2022	[147]
E28	EC	EIS	Boron doped diamond (BDD)	Antibody	N	PBS	4.93 × 10^–12^ (0.227 ng/mL)*	9.6 × 10^–14^ to 9.6 × 10^–11^	3	–					AUG 2022	[148]
E29	EC	EIS	–	Antibody	S1	Buffer	2.93 × 10^–12^ (0.23 ng/mL)*	6.4 × 10^–12^ to 1.3 × 10^–10^	1	–					AUG 2022	[120]
						Artificial Saliva	1.15 × 10^–12^ (0.09 ng/mL)*	1.3 × 10^–11^ to 1.3 × 10^–10^	1							
E30	EC	EIS	MoS2-PDA nanosheet	Antibody	N	0.1 M PBS	5.83 × 10^–20^ (2.8 ag/mL)*	2.1 × 10^–19^ to 2.1 × 10^–9^	10	–					SEP 2022	[149]
E31	EC	EIS	–	Aptamer	S(RBD)	0.01 M PBS	1.57 × 10^–14^ (0.4 pg/mL)*	3.9 × 10^–15^ to 5.0 × 10^–13^	2	–					OCT 2022	[150]
F01	EC	OECT	–	Engineered Antibody	S(RBD)	Buffer	4.80 × 10^–14^	Not determined	–	–					MAY 2021	[151]
						Saliva	2.30 × 10^–14^									
					S1	Buffer	1.80 × 10^–20^	Attomolar to Nanomolar range	–							
						Saliva	1.20 × 10^–21^									
F02	EC	OECT	–	Antibody	S(RBD)	PBS	3.64 × 10^–16^ (10 fg/mL)*	Not determined	–	–					NOV 2021	[152]
G01	EC	PEC	Metal–organic frameworks (MOF)	Aptamer	S (S1 + S2)	N/A	5.36 × 10^–10^ (72 ng/mL)*	3.7 × 10^–9^ to 5.9 × 10^–8^	1	–					OCT 2021	[153]
G02	EC	PEC	Palladium nanoparticles	Antibody	S (S1 + S2)	PBS	7.14 × 10^–18^ (1 fg/mL)*	7.1 × 10^–18^ to 7.1 × 10^–11^	6	–					MAY 2022	[154]
G03	EC	PEC	Graphitic carbon nitride (gC3N4) and cadmium sulfide (CdS) quantum dots	Aptamer	S(RBD)	N/A	1.20 × 10^–10^	5.0 × 10^–10^ to 3.2 × 10^–8^	1	–					OCT 2021	[155]
G04	EC	PEC	Au@TiO_2_	Engineered Antibody	S1	PBS	6.36 × 10^–17^ (5 fg/mL)*	1.9 × 10^–16^ to 1.9 × 10^–10^	6	–					JUN 2022	[156]
G05	EC	PEC	Dioxide@bismuth tungstate nanocomposite	Antibody	N	N/A	8.26 × 10^–15^ (0.38 pg/mL)*	2.2 × 10^–14^ to 1.1 × 10^–12^	1	–					AUG 2022	[157]
H01	EL	FET	Graphene	Antibody	S1	0.001 × PBS	1.05 × 10^–13^ (8 pg/mL)*	1.0 × 10^–13^ to 1.0 × 10^–9^	5	–					MAR 22020	[158]
				ACE-2	S1	0.001 × PBS	1.96 × 10^–13^ (15 pg/mL)*	1.0 × 10^–12^ to 1.0 × 10^–8^	4							
H02	EL	FET	Graphene	Antibody	S1	PBS	1.31 × 10^–17^ (1 fg/mL)*	1.3 × 10^–18^ to 1.3 × 10^–11^	4	Inactivated virus	Culture medium	1.6 × 10^1^ (PFU/mL)	1.6 × 10^1^ to 1.6 × 10^4^ (PFU/mL)	3	APR 2020	[159]
				Antibody	S1	0.01X VTM	1.31 × 10^–15^ (100 fg/mL)*	1.3 × 10^–15^ to 1.3 × 10^–12^	3							
H03	EL	FET	Single-walled carbon nanotube (SWCNT)	Antibody	S1	PBS	7.19 × 10^–18^ (0.55 fg/mL)*	7.2 × 10^–18^ to 7.2 × 10^–7^	11	–					FEB 2021	[43]
				Antibody	N	PBS	3.33 × 10^–19^ (0.016 fg/mL)*	3.3 × 10^–19^ to 3.3 × 10^–7^	12							
H04	EL	FET	MXenes/graphene	Antibody	S1	PBS	1.31 × 10^–17^ (1 fg/mL)*	1.3 × 10^–17^ to 1.3 × 10^–11^	6	–					MAR 2021	[160]
H05	EL	FET	TMCs	Antibody	S1	PBS	3.27 × 10^–16^ (25 fg/mL)*	2.6 × 10^–15^ to 1.0 × 10^–10^	4	–					JUN 2021	[161]
H06	EL	FET	Semiconducting polymer	Antibody	S(RBD)	PBS	2.71 × 10^–12^ (74.6 pg/mL)*	3.6 × 10^–13^ to 3.6 × 10^–7^	6	–					AUG 2021	[162]
H07	EL	FET	Reduced graphene oxide (rGO)	Antibody	S1	1X PBS	2.00 × 10^–18^	2.0 × 10^–18^ to 2.0 × 10^–11^	5	–					SEP 2021	[163]
H08	EL	FET	Carbon nanotube (CNT)	Antibody	S1	10 mM AA	5.39 × 10^–17^ (4.12 fg/mL)*	1.3 × 10^–18^ to 6.5 × 10^–14^	4	–					OCT 2021	[164]
H09	EL	FET	Crumbled graphene	Antibody	S1	0.1X PBS	1.00 × 10^–18^	1.0 × 10^–18^ to 1.0 × 10^–9^	9	–					NOV 2021	[165]
				Antibody	N	1X PBS	1.00 × 10^–17^	1.0 × 10^–18^ to 1.0 × 10^–10^	8							
H10	EL	FET	Graphene	Antibody	S1	Artificial Saliva	4.45 × 10^–19^	1.0 × 10^–18^ to 1.0 × 10^–11^	7	–					NOV 2021	[73]
H11	EL	FET	–	ACE-2	S1	1X PBS	1.31 × 10^–17^ (1 fg/mL)*	1.3 × 10^–17^ to 1.3 × 10^–9^	8	Synthetic virus	PBS	165 (copies/mL)	1.7 × 10^0^ to 1.7 × 10^4^ (copies/mL)	4	NOV 2021	[166]
H12	EL	FET	Graphene oxide (GO)/graphene	Antibody	S1	1X PBS	1.02 × 10^–16^ (8 fg/mL)*	1.3 × 10^–16^ to 1.3 × 10^–12^	4	–					DEC 2021	[167]
H13	EL	FET	–	Antibody	N	1X PBS	7.44 × 10^–12^ (0.34 ng/mL)*	8.7 × 10^–12^ to 8.7 × 10^–9^	3	–					DEC 2021	[168]
				Antibody	N	Artificial Saliva	2.96 × 10^–12^ (0.14 ng/mL)*	8.7 × 10^–12^ to 8.7 × 10^–9^	3							
H14	EL	FET	Graphene	Antibody	S1	0.1X HEPES	7.40 × 10^–10^	1.0 × 10^–10^ to 1.0 × 10^–7^	3	–					JAN 2022	[169]
					Ferritin	0.1X HEPES	2.30 × 10^–10^	Not determined	–							
H15	EL	FET	Boron and nitrogen co-doped graphene oxide (GO) Gel	Antibody	N	Buffer	2.17 × 10^–20^ (10 ag/mL)*	2.2 × 10^–20^ to 2.2 × 10^–8^	12	–					APR 2022	[170]
H16	EL	FET	Reduced graphene oxide (rGO)	Antibody	S1	PBS	4.33 × 10^–14^ (3.4 pg/mL)*	4.3 × 10^–14^ to 4.3 × 10^–10^	4	–					MAY 2022	[171]
						Saliva-relevant	Few pg/mL	6.4 × 10^–15^ to 6.4 × 10^–8^	7							
H17	EL	FET	Graphene	Antibody	S(RBD)	–	–	Femtomolar to nanomolar range	–	Inactivated virus	1X PBS (with 0.5 mM MgCl^2^)	1.28 (PFU/mL)	6.68 × 10^0^ to 6.68 × 10^4^ (PFU/mL)	4	JUN 2022	[172]
					N	–	–	Femtomolar to nanomolar range	–			1.45 (PFU/mL)	6.68 × 10^0^ to 6.68 × 10^4^ (PFU/mL)	4		
H18	EL	FET	Graphene	Aptamer	S1	0.001 × PBS buffer	3.00 × 10^–15^	3.3 × 10^–15^ to 3.3 × 10^–9^	6	–					JUN 2022	[173]
H19	EL	FET	Graphene	Antibody	S1	50 mM PB	1.00 × 10^–14^	1.0 × 10^–15^ to 1.0 × 10^–6^	9	–					JUL 2022	[174]
I01	EL	DIDC	Graphene oxide (GO)	Antibody	S1	0.1 M PBS	1.27 × 10^–17^ (1 fg/mL)*	1.3 × 10^–17^ to 1.3 × 10^–5^	12	–					AUG 2021	[175]
I02	EL	Tri-channel transistor	In_2_O_3_/ZnO	Antibody	S1	0.1X PBS	8.65 × 10^–16^	1.0 × 10^–16^ to 1.0 × 10^–6^	10	–					NOV 2021	[176]
I03	EL	Capacitive biosensor	Epitaxial graphene	Antibody	S1	1X PBS	1.31 × 10^–20^ (1 ag/mL)*	1.0 × 10^–20^ to 1.0 × 10^–8^	12	Inactivated virus	N/A	60 (copies/mL)	6.0 × 10^1^—2.5 × 10^2^ (copies/mL)	–	NOV 2021	[177]
I04	EL	Capacitive biosensor	–	ACE-2	S1	PBS	8.07 × 10^–8^ (750 pg/μL/mm^2^)*	1.3 × 10^–9^ to 1.3 × 10^–7^	2	–					JAN 2022	[178]
I05	EL	Nanowire array biosensor	Silicon nanowire (SiNWs)	ACE-2	S1	0.1X PBS	5.75 × 10^–10^ (100 ng/mL)*	5.8 × 10^–10^ to 5.8 × 10^–8^	2	–					MAR 2022	[179]
I06	EL	2D MoS_2_ sensor array	Amorphous MoS_2_	Engineered Antibody	S1	1X PBS	2.54 × 10^–14^ (2 pg/mL)*	2.5 × 10^–14^ to 2.5 × 10^–10^	4	–					MAR 2022	[180]
J01	OP	FL	Carbon nanotube (CNT)	Antibody	S(RBD)	PBS	1.26 × 10^–8^	Not determined	–	–					FEB 2021	[62]
J02	OP	FL	Graphene oxide quantum dots (GOQDs)	Antibody	S1	PBS	2.93 × 10^–15^ (0.23 pg/mL)*	1.3 × 10^–14^ to 1.3 × 10^–9^	5	–					JUL 2021	[181]
					N	PBS	7.29 × 10^–15^ (0.35 pg/mL)*	2.1 × 10^–14^ to 2.1 × 10^–9^	5							
J03	OP	NIR	Carbon nanotube (CNT)	–	S1	Buffer	3.50 × 10^–10^	1.3 × 10^–15^ to 1.3 × 10^–8^	7	–					SEP 2021	[182]
					N	Buffer	4.80 × 10^–14^	2.2 × 10^–15^ to 2.2 × 10^–8^	7							
J04	OP	FL	Magnetic beads	Antibody	N	PBST	6.93 × 10^–13^ (33.28 pg/mL)*	2.1 × 10^–12^ to 2.1 × 10^–8^	4	–					SEP 2021	[183]
J05	OP	FL	Au@Pt nanoparticles	Antibody	N	0.1 M PBS (2% T20)	5.42 × 10^–13^ (0.026 ng/mL)*	1.0 × 10^–12^ to 3.3 × 10^–11^	1	–					SEP 2021	[184]
J06	OP	FL	–	Aptamer	S1	N/A	2.70 × 10^–10^ (21 ng/mL)*	4.2 × 10^–11^ to 6.5 × 10^–7^	4	–					NOV 2021	[185]
J07	OP	FL	Au nanoparticles (AuNPs)	Aptamer	N	Phosphate Buffer	3.26 × 10^–15^ (150 fg/mL)*	8.7 × 10^–15^ to 4.5 × 10^–13^	1	–					OCT 2022	[186]
K01	OP	CL	Co–Fe@hemin	Antibody	S(RBD)	Buffer	3.77 × 10^–12^ (0.1 ng/mL)*	7.5 × 10^–12^ to 3.8 × 10^–9^	2	–					NOV 2020	[187]
K02	OP	CL	Au nanoparticles (AuNPs)	Antibody	N	PBS	1.44 × 10^–15^ (69 fg/mL)*	2.1 × 10^–15^ to 2.1 × 10^–10^	5	–					OCT 2021	[188]
L01	OP	SPR	Graphene oxide (GO)	Aptamer	N	FBS	6.25 × 10^–18^	1.0 × 10^–18^ to 1.0 × 10^–11^	7	–					APR 2021	[189]
			Graphene oxide (GO)	Aptamer	N	FBS	6.25 × 10^–19^	1.0 × 10^–19^ to 1.0 × 10^–7^	12							
L02	OP	SPR	Graphene	Antibody	S(RBD)	500 nM PBS	1.95 × 10^–9^	2.0 × 10^–9^ to 6.3 × 10^–8^	1	–					MAY 2021	[190]
L03	OP	SPR	Au nanoparticles (AuNPs)	Aptamer	S1	PBS	1.60 × 10^–8^	6.3 × 10^–8^ to 2.5 × 10^–7^	1	–					SEP 2021	[191]
L04	OP	SPR	Au nanoparticles (AuNPs)	Antibody	N	Buffer	8.50 × 10^–14^	2.2 × 10^–13^ to 2.2 × 10^–10^	3	–					JAN 2022	[192]
L05	OP	SPR	–	Antibody	S1	DPBS	1.02 × 10^–15^ (0.08 pg/mL)*	1.3 × 10^–13^ to 1.3 × 10^–8^	5	–					MAR 2022	[193]
L06	OP	SPR	Ti_3_C_2_-MXene nanosheet	Antibody	S1	PBS	1.53 × 10^–16^ (12 fg/mL)*	1.3 × 10^–14^ to 1.3 × 10^–7^	7	–					MAY 2022	[194]
L07	OP	SPR	–	Engineered antibody	S(RBD)	N/A	1.00 × 10^–8^	Not determined	–	Inactivated virus					JUL 2022	[195]
M01	OP	LSPR	Au nanoparticles (AuNPs)	Antibody	N	PBS	3.13 × 10^–9^ (150 ng/mL)*	3.1 × 10^–9^ to 1.4 × 10^–8^	< 1	–					SEP 2021	[196]
M02	OP	LSPR	Silver nanotriangle array	ACE-2	S(RBD)	PBS	8.30 × 10^–10^	2.0 × 10^–12^ to 9.4 × 10^–9^	3	CoV-NL63 virus	PBS	3.9 × 10^2^ (PFU/mL)	3.9 × 10^2^ to 1.0 × 10^5^ (PFU/mL)	2	FEB 2022	[197]
											PBS	6.3 × 10^2^ (PFU/mL)	6.3 × 10^2^ to 1.0 × 10^4^ (PFU/mL)	1		
M03	OP	LSPR	Au nanoparticles (AuNPs)	MIP	S1 (α)	PBS/serum	9.71 × 10^–15^	1.0 × 10^–13^ to 1.0 × 10^–7^	6	–					APR 2022	[198]
					S1 (β)		7.32 × 10^–15^									
					S1 (γ)		8.81 × 10^–12^									
N01	OP	Fiber optics (SPR)	–	MIP	S1	42 nM PBS	5.80 × 10^–8^	6.5 × 10^–8^ to 6.5 × 10^–6^	2	–					MAR 2021	[199]
N02	OP	Fiber optics (SPR)	–	Aptamer	S (S1 + S2)	Buffer	3.70 × 10^–8^	2.5 × 10^–8^ to 1.0 × 10^–6^	1	–					MAY 2021	[200]
N03	OP	Fiber optics (SPR)	–	Peptide	Protease	Serum	1.00 × 10^–12^	3.3 × 10^–12^ to 1.0 × 10^–8^	3	–					JUN 2021	[63]
N04	OP	Fiber Optics	Au nanoparticles (AuNPs)	Antibody	S1	PBS	1.31 × 10^–14^ (1 pg/mL)*	1.3 × 10^–14^ to 1.3 × 10^–6^	8	–					AUG 2021	[201]
N05	OP	Fiber optics (FL)	Polystyrene microspheres	Antibody	N	PBS	1.63 × 10^–13^ (7.5 pg/mL)*	1.7 × 10^–13^ to 2.2 × 10^–11^	2	–					JUN 2022	[202]
N06	OP	Fiber optics (BLI)	–	Antibody	S1	Buffer	1.25 × 10^–11^	1.3 × 10^–11^ to 4.0 × 10^–10^	1	–					MAY 2022	[203]
					S(RBD)	Buffer	3.60 × 10^–11^	3.6 × 10^–11^ to 7.2 × 10^–10^	1							
O01	OP	SERS	Au nanoparticles (AuNPs)	Aptamer	S1	PBS	1.00 × 10^–15^	1.0 × 10^–12^ to 1.0 × 10^–6^	6	–					FEB 2021	[69]
O02	OP	SERS	Au nanoparticles (AuNPs)	Antibody	S1	Saliva	7.94 × 10^–17^ (6.07 fg/mL)	1.3 × 10^–16^ to 1.3 × 10^–10^	6	–					JUN 2021	[204]
						Serum	9.94 × 10^–17^ (7.60 fg/mL)	Not determined	–							
						Blood	1.31 × 10^–15^ (0.10 pg/mL)	not determined	–							
						PBS	1.01 × 10^–17^ (0.77 fg/mL)	1.3 × 10^–17^ to 1.3 × 10^–11^	6							
O03	OP	SERS	Au nanostar	–	S1	Buffer	8.89 × 10^–9^	7.4 × 10^–4^ to 7.0 × 10^–9^	5	–					FEB 2021	[205]
					N	Buffer	Not determined	Not determined	–							
O04	OP	SERS	Carbon nanotube (CNT)	Engineered antibody	S(RBD)	PBS	Not determined	Not determined	–	Inactivated virus	PBS	17 (virus/μL)	20 to 20,000 (virus/μL)	3	JUL 2021	[206]
O05	OP	SERS	Au nanoparticles (AuNPs)	Antibody	S1	Tris buffer	3.00 × 10^–7^	Not determined	–	–					AUG 2021	[207]
O06	OP	SERS	Au nanoparticles/silicon nanowire (AgNPs/SiNW)	–	S(RBD)	PBS	9.30 × 10^–12^	9.3 × 10^–12^ to 9.3 × 10^–6^	6	–					SEP 2021	[208]
O07	OP	SERS	Au nanoparticles (AuNPs)	Aptamer	S(RBD)	Mixed protein	2.50 × 10^–11^ (0.625 ng/mL)*	2.5 × 10^–11^ to 4.0 × 10^–10^	1	–					OCT 2021	[209]
						Urine	5.00 × 10^–11^ (1.25 ng/mL)*	5.0 × 10^–11^ to 8.0 × 10^–10^	1							
						Blood	5.00 × 10^–11^ (1.25 ng/mL)*	5.0 × 10^–11^ to 8.0 × 10^–10^	1							
O08	OP	SERS	Au nanoparticles (AuNPs)	Antibody	N	PBS	5.33 × 10^–17^ (2.56 fg/mL)*	2.1 × 10^–16^ to 2.1 × 10^–11^	5	Inactivated virus (lysate)	N/A	3.4 (PFU/mL)	1.0 × 10^0^ to 1.0 × 10^3^ (PFU/mL)	3	JAN 2022	[210]
O09	OP	SERS	3D mag-MoO3-PDA@Au NS	ACE-2	S1	PBS	5.73 × 10^–17^ (4.5 fg/mL)*	1.3 × 10^–16^ to 1.3 × 10^–11^	5	–					JAN 2022	[211]
						Cell Lysate	1.23 × 10^–16^ (9.7 fg/mL)*	1.3 × 10^–16^ to 1.3 × 10^–11^	5							
						Saliva	1.14 × 10^–7^ (3.13 ug/mL)*	1.1 × 10^–7^ to 1.8 × 10^–6^	1							
O10	OP	SERS	Magnetic nanoparticles	Engineered antibody	S1	PBS	3.27 × 10^–15^ (257 fg/mL)*	1.3 × 10^–14^ to 1.3 × 10^–10^	4	Inactivated virus	VTM	4.1 × 10^4^ (genomes/mL)	1.3 × 10^5^ to 1.3 × 10^9^ (genomes/mL)	4	MAR 2022	[212]
O11	OP	SERS	Core–shell Au@Silica nanoparticles	Antibody	S1	N/A	6.05 × 10^–13^(0.046 ng/mL)*	1.3 × 10^–13^ to 1.3 × 10^–9^	4	–					JUL 2022	[213]
O12	OP	SERS	Core–shell Au nanoparticles (AuNPs)	Antibody	S(RBD)	PBS	7.10 × 10^–16^ (19.2 fg/mL)*	3.7 × 10^–15^ to 3.7 × 10^–8^	7	–					AUG 2022	[214]
P01	OP	Colorimetric	Au nanoparticles (AuNPs)	ACE-2	S1	0.1 M PBS	1.96 × 10^–15^ (0.154 pg/mL)*	1.3 × 10^–14^ to 1.3 × 10^–8^	6	–					OCT 2021	[215]
P02	OP	Colorimetric	Core–shell Au@Pt nanoparticles	Antibody	S1	N/A	1.40 × 10^–10^ (11 ng/mL)*	1.3 × 10^–10^ to 1.3 × 10^–9^	1	–					DEC 2021	[216]
P03	OP	Colorimetric	Core–shell Au@Pt nanoparticles	Antibody	N	PBS	1.27 × 10^–15^ (0.1 pg/mL)*	2.2 × 10^–15^ to 2.2 × 10^–11^	4	–					AUG 2022	[217]
Q01	OP	Plasmonic metasensor	Au nanoparticles (AuNPs)	Antibody	S1	PBS	4.20 × 10^–15^	Not determined	–	–					JAN 2021	[218]
Q02	OP	Phononic sensor	Graphene	Antibody	S(RBD)	PBS	2.60 × 10^–18^ (1.02 fg/mL)*	2.6 × 10^–14^ to 2.6 × 10^–7^	7	–					JUN 2021	[219]
						Artificial saliva	9.60 × 10^–18^ (3.75 fg/mL)*	1.3 × 10^–17^ to 1.0 × 10^–16^	–							
R01	ME	Micro cantilever	–	Antibody	S(RBD)	PBS	3.30 × 10^–11^	3.3 × 10^–11^ to 3.3 × 10^–8^	3	inactivated virus	Lysis buffer	100 (copies/mL)	1.0 × 10^2^ to 6.0 × 10^9^ (copies/mL)	7	SEP 2021	[220]
					N		2.08 × 10^–11^	2.1 × 10^–11^ to 2.1 × 10^–8^	3							
S01	GR	MPS	Magnetic nanoparticles	Antibody	S1	PBS	1.56 × 10^–9^	Not determined	–	–					SEP 2021	[221]
					N	PBS	1.25 × 10^–8^	Not determined	–							
T01	TH	Thermal assay	–	MIP	S(RBD) (α)	PBS	2.40 × 10^–16^ (6.1 fg/mL)*	3.6 × 10^–17^ to 3.6 × 10^–13^	4	–					APR 2022	[222]
					S(RBD) (γ)	PBS	3.60 × 10^–16^ (9.9 fg/mL)*	3.6 × 10^–17^ to 3.6 × 10^–13^	4							
U01	CO	SWV (+ LFA)	Graphene oxide (GO)	Antibody	S(RBD)	N/A	4.40 × 10^–12^ (0.11 ng/mL)*	1.3 × 10^–11^ to 1.3 × 10^–8^	3	–					DEC 2020	[223]
U02	CO	SERS	Porous graphene oxide (GO)	Antibody	S1	PBS	7.50 × 10^–14^	1.0 × 10^–12^ to 1.0 × 10^–7^	4	–					JUL 2021	[224]
		SWV					3.90 × 10^–14^	5.0 × 10^–13^ to 1.0 × 10^–7^	5							
U03	CO	LSPR	Au nanoparticles (AuNPs)	Antibody	S1	PBS	1.27 × 10^–14^ (1 pg/mL)*	1.3 × 10^–14^ to 1.3 × 10^–10^	4	–					OCT 2021	[225]
		SWV				PBS	6.11 × 10^–10^ (48 ng/mL)*	–	–							
U04	CO	LFA	Au nanoparticles (AuNPs)	Antibody	S(RBD)	PBST	4.63 × 10^–11^ (1.2 ng/mL)*	1.6 × 10^–10^ to 2.3 × 10^–9^	1	–					DEC 2021	[226]
		SERS				PBST	3.86 × 10^–12^ (0.1 ng/mL)*	3.9 × 10^–12^ to 3.9 × 10^–10^	2							
U05	CO	TCA (+ LFA)	Au nanoparticles (AuNPs)	Antibody	S(RBD)	Buffer	4.50 × 10^–19^	–	–	–					DEC 2021	[227]
						NP Wash	3.60 × 10^–18^	1.3 × 10^–17^ to 1.0 × 10^–15^	1							
U06	CO	SERS (+ LFA)	Au nanoparticles (AuNPs)	ACE-2	S(RBD)	Buffer	2.84 × 10^–8^ (0.78 ug/mL)*	–	–	–					JAN 2022	[228]
						Saliva	1.14 × 10^–7^ (3.13 ug/mL)*	1.1 × 10^–7^ to 1.8 × 10^–6^	1	–						

* The original data from each article are provided in parentheses without converting to molar concentration

^a^*PBS* Phosphate Buffered Solution, *PBST* PBS buffer with Tween 20, *DPBS* Dulbecco’s Phosphate-Buffered Saline, HEPES: Hydroxyethyl piperazine Ethane Sulfonicacid, *AA* l-ascorbic acid, *FBS* Fetal Bovine Serum, *NP* Nasopharyngeal, *N/A* not available

^b^*VTM* Viral Transport Medium, *UTM* Universal Transport Medium, *VTMT* VTM with Tween 80 and Igepal

In terms of clinical sensitivity, the most significant and practical guideline can be established by referencing the current gold standard method, RT-PCR. The performance of the developed nanobiosensor needs to be as sensitive as RT-PCR, considered the last and final procedure for evaluating clinical samples in numerous studies because important indices, like clinical sensitivity, clinical specificity, and clinical accuracy, can be calculated. Researchers collected positive and negative samples confirmed by RT-PCR and compared the results obtained from developed sensors. A total of 14.6% (23/158) of articles presented clinical validation, and the results are summarized in Table 3. The results vary from 53.6% (H03) to 100.0% (A02, A05, A09, A14, C03, E05, E21) according to the detection technique, design of the nanobiosensors, and the characteristics of the clinical samples.

**Table 3:** The performance of the nanobiosensors in the test using clinical samples

ID	Design					Clinical samples					Performance evaluation								Note
											head-to-head comparison				Agreement with RT-PCR				
	Transduction mechanism	Detection technique	Materials/nanomaterials	Biorecognition element	Target antigen	Source^a^	Dilution	Total	Positive	Negative	RT-PCR^b^	Proposed sensor	ELISA	RAT	Case	Clinical sensitivity (%)	Clinical specificity (%)	Clinical accuracy (%)	
A02	EC	DPV	Carbon Black	Antibody	S1	Saliva	No	24	7	17	24/24	22/24	–	–	24	100.0	88.2	91.7	[83]
					N	Saliva	No	20	6	14	20/20	18/20	–	–	20	83.3	92.9	90.0	
A05	EC	DPV	Laser-scribed Graphene	Aptamer	S(RBD)	Serum	0.1 M PBS w/0.1 M KCl	23	17	6	23/23	23/23	12/23 (IgG)	–	23	100.0	100.0	100.0	[86]
													14/23 (IgM)						
A06	EC	DPV	Graphene Oxide	Antibody	S1	Gargle & Mouthwash	Lysis Buffer	110	30	80	12/12	11/12	–	8/12	110	93.3	92.5	92.7	[87]
A09	EC	DPV	AuNPs	ACE-2	S1	Saliva	PBS (1:1000)	32	16	16	32/32	31/32	–	–	32	100.0	93.8	96.9	[90]
A10	EC	DPV	AuNPs	Antibody	S1	NP (in VTM)	Dilution	17	6	11	17/17	14/17	–	–	17	66.7	90.9	82.4	[91]
A14	EC	DPV	Au Nanorod	Antibody	S(RBD)	NP (in VTM)	N/A	10	5	5	10/10	8/10	–	–	10	100.0	60.0	80.0	[95]
A15	EC	DPV	AuNPs	Antibody	S1	NP (in VTM)	N/A	37	19	18	37/37	36/37	–	–	37	94.7	100.0	97.3	[96]
B06	EC	SWV	AuNPs	ACE-2	S1	NP (in VTM)	N/A	103	53	50	103/103	90/103	–	–	103	88.7	86.0	87.4	[105]
						Saliva	N/A	10	3	7	10/10	10/10	–	–	10	100.0	100.0	100.0	
B11	EC	SWV	Graphene Oxide (GO)	Antibody	N	NP (in Detergent Solution)	N/A	60	30	30	60/60	53/60	–	–	60	90.0	86.7	88.3	[110]
					N (Ο)	NP (in Detergent Solution)	N/A	50	25	25	50/50	47/50	–	–	50	96.0	92.0	94.0	
C03	EC	CA	–	Antibody	S1	Serum (INF A)^c^	N/A	79	42	37	79/79	77/79	–	–	79	100.0	94.6	97.5	[115]
C06	EC	CA	–	Antibody	N	NP (in VTM)	VTMT	37	28	9	37/37	28/37	–	–	37	67.9	100.0	75.7	[118]
D02	EC	POT	–	Cell	S1	NP (in TM)	0.9% saline solution (in PBS)	24	14	10	24/24	23/24	–	–	24	92.9	100.0	95.8	[122]
D03	EC	POT	–	Cell	S1	NP (in TM)	PBS	17	7	10	17/17	17/17	–	–	17	100.0	100.0	100.0	[123]
E05	EC	EIS	–	ACE-2	S1	NP/OP (in TM)	PBS	139	109	30	139/139	121/139	–	–	139	83.5	100.0	87.1	[76]
						Saliva	PBS	50	13	37	50/50	45/50	–	–	50	100.0	86.5	90.0	
E21	EC	EIS	Magnetic Nanoparticle	Antibody	S1	NP (in vNAT buffer)	PBS (1:100)	50	40	10	50/50	46/50	–	41/50	50	90.0	100.0	92.0	[141]
				Antibody	S2			50	40	10	50/50	45/50			50	87.5	100.0	90.0	
				Antibody Cocktail	S1/S2			50	40	10	50/50	50/50			50	100.0	100.0	100.0	
H03	EL	FET	SWCNT	Antibody	S1	NP (in VTM)	N/A	38	28	10	38/38	30/38	–	–	38	82.1	70.0	78.9	[43]
					N		N/A	38	28	10	38/38	22/38	–	–	38	53.6	70.0	57.9	
H10	EL	FET	Graphene	Antibody	S1	NP (in UTM)	N/A	25	14	11	25/25	24/25	–	–	25	92.9	100.0	96.0	[73]
J05	OP	FL	Au@PtNPs	Antibody	N	Blood	PBST	101	21	80	101/101	96/101	90/101	–	101	76.2	100.0	95.0	[184]
J06	OP	FL	–	Aptamer	S1	NP (in VTM)	N/A	50	45	5	50/50	38/50	–	–	50	80.0	25.0	75.5	[185]
L07	OP	SPR	–	Engineered Antibody	S(RBD)	NP (in VTM)	N/A	119	50	69	119/119	108/119	–	–	119	88.0	92.8	90.8	[195]
N05	OP	FO	Polystyrene Microspheres	Antibody	N	Serum/Plasma	No	125	25	100	125/125	118/125	117/125	–	125	72.0	100.0	94.4	[202]
P01	OP	COL	Au Nanoparticles (AuNPs)	ACE-2	S1	NP (in VTM)	N/A	100	50	50	100/100	90/100	–	–	100	96.0	84.0	90.0	[215]
R01	ME	MC	Magnetic Nanoparticle	Antibody	S1/N	NP (in TM)	N/A	11	9	2	11/11	10/11	–	–	11	88.9	100.0	90.9	[220]

^a^NP: Nasopharyngeal (Swab); OP: Oropharyngeal (Swab)

^b^The clinical samples are usually verified by RT-PCR

^c^C03 utilized clinical samples obtained from Influenza-infected patients as a model SARS-CoV-2 samples

#### Specific tests (S)

In addition to sensitive detection, diagnostic tests are also required to reach a low false positive rate, where the tests should not misdiagnose healthy individuals or patients with other infections as COVID-19 patients. From a commonsense standpoint, a false positive diagnosis might cause an unnecessary medical procedure and additional medical expenses. More importantly, a prescription of the wrong medicine or an order of the wrong treatment can put people at risk. However, COVID-19 cases seem to need a different approach from others. Due to the aggressive spreading of this highly infectious virus, the primary measure for the just-diagnosed individual is usually quarantine. Except for the older age group who are hospitalized with underlying diseases, other infected individuals at the very early stage take common drugs, like fever reducers. Therefore, there is more leeway regarding false positives compared to other viral diseases since the outcome of the overdiagnosis is not relatively fatal. In contrast, missing the infected with false negatives could be more dangerous to the entire society. Nevertheless, the importance of specific detection should not be simply overruled and is related to the accuracy and reliability of the developed tests. The low specificity of the diagnostics also endangers the entire healthcare system with enormous socioeconomic burdens due to the unnecessary quarantine accompanied by repetitive tests to end quarantine.

Without clinical samples, the specificity of the developed nanobiosensors can be tested using model samples containing other non-target antigens (Table 4). Almost 66.5% (105/158) of the nanobiosensor studies provided related data under various sample compositions. Model samples were mimicked using non-target SARS-CoV-2 structural proteins, other beta-coronaviruses (SARS-CoV and MERS-CoV), human coronaviruses (HCoV-HKU1, HCoV-NL63, HCoV-OC43, and HCoV-229E), animal coronaviruses (BCoV and FCoV), and other viral proteins (Influenza A, Influenza B, mouse hepatitis virus, herpes simplex virus, vesicular stomatitis virus, parainfluenza virus, adenovirus, varicella-zoster virus, and rubella virus). Specificity testing with model samples is also an important validation because the viral proteins may share a homology among their families, and sometimes, the detection results may be indistinguishable from phylogenetically similar viruses. For example, in the case of SARS-CoV-2, both S and N proteins showed 76% and 90% sequence similarity with S and N proteins of SARS-CoV. In fact, several studies have pointed out that a certain amount of signal was detected from the SARS-CoV antigen [81]. However, non-viral proteins were also utilized to prove that the performance of the nanobiosensor was not inhibited by those proteins (bovine serum albumin, human serum albumin, CD48, HER-2, IL-6, Beta-lactamase, and others). Some studies designed the experiment considering the effect of the interfering agents (especially in electrochemical detection) and further application with biological fluids.

**Table 4:** The design of specificity and selectivity tests in the nanobiosensor research

ID	Design					Specificity and selectivity tests							References
	Transduction mechanism	Detection technique	Nanomaterials/materials	Biorecognition element	Target antigen	Viral protein					Non-viral protein, small molecule, and bacterium^b^	Total	
						SARS-CoV-2 protein	Other coronavirus protein			Other viral protein^a^			
							Beta-coronavirus	Human coronavirus	Animal coronavirus				
A02	EC	DPV	Carbon black	Antibody	S1	–	–	–	–	IFV A	–	2	[83]
										IFV H1N1			
					N	–	–	–	–	–	–		
A03	EC	DPV	–	MIP	N	SARS-CoV-2 S	–	–	–	HCV E2	BSA	4	[84]
											CD48		
A07	EC	DPV	Au nanoparticles (AuNPs)	Antibody	S1	–	MERS-CoV S	–	–	AIV	–	3	[88]
										HIV			
A08	EC	DPV	Au/graphene	MIP	N	SARS-CoV-2 S	–	–	–	–	BSA	5	[89]
											CD48		
											HER-2		
											MPT64		
											Cardiac Troponin (cTnl)		
A11	EC	DPV	g-C3N4/Au/WO3	Antibody	N	–	MERS-CoV N	–	–	IFV H1N1	–	2	[92]
A14	EC	DPV	Au nanorod	Antibody	S(RBD)	SARS-CoV-2 S		–	–	–	–	1	[95]
A15	EC	DPV	Au nanoparticles (AuNPs)	Antibody	S1	SARS-CoV-2 S	–	–	–	IFV H1N1	–	2	[96]
A16	EC	DPV	Laser-scribed graphene (LSG)	ACE-2	S1			HCoV-NL63 N		IFV H1N1		7	[171]
										IFV B			
										HPV			
								HCoV-229E N		RSV			
										HRV			
A17	EC	DPV	Au nanoparticles (AuNPs)	Aptamer	S1						BSA	2	[98]
											HSA		
A18	EC	DPV	Single-walled carbon nanotube (SWCNT)	Aptamer	S(RBD)		MERS-CoV S					2	[99]
A19	EC	DPV	Ag/reduced graphene oxide	Antibody	S(RBD)		SARS-CoV S					3	[100]
B01	EC	SWV	Carbon nanofiber	Antibody	N	–	–	HCoV HKU1	–	–	–	1	[101]
B02	EC	SWV	Graphene	Antibody	S1	–	–	–	–	–	Beta-lactamase	1	[102]
B03	EC	SWV	Au nanoparticles (AuNPs)	Antibody	N	–	MERS-CoV N	HCoV HKU1	–	IFV A	–	4	[75]
										IFV B			
B05	EC	SWV	Au cluster	Antibody	S1	–	–	–	–	IFV A	–	2	[104]
										IFV B			
B06	EC	SWV	Au nanoparticles (AuNPs)	ACE-2	S1	–	–	–	–	INF A (H1N1)	–	4	[105]
										IFV B			
										HSV			
										MHV			
B07	EC	SWV	–	Aptamer	S1	–	SARS-CoV S	–		NGAL	–	3	[106]
							MERS-CoV N						
B09	EC	SWV	–	MIP	S1	–	SARS-CoV N	–	–	HCV E2	HAS	4	[108]
											IgG		
B10	EC	SWV	Laser-engraved graphene	Aptamer	S(RBD)	–	–	–	–	IFV A	C-reactive protein	3	[109]
										Hemagglutin (HA)			
B11	EC	SWV	Au nanoparticles (AuNPs)	Antibody	N	–	–	–	–	INF A INF B	PSA	5	[110]
											AFP		
											CEA		
B12	EC	SWV	Single-walled carbon nanotube (SWCNT)	Antibody	S1	–	–	–	–	INF A	–	2	[111]
										INF B			
C02	EC	CA	–	Antibody	S1	–	SARS-CoV S	HCoV-NL63	BCoV	RuV	–	6	[114]
							MERS-CoV S			EBOV			
C03	EC	CA	–	Antibody	S1	–	SARS-CoV S	HCoV-NL63	–	–	–	3	[115]
							MERS-CoV S						
C04	EC	CA	Magnetic nanobeads	Antibody	N	SARS-CoV-2 S(RBD)	SARS-CoV N	–	–	–	–	3	[116]
					N		MERS-CoV N						
C05	EC	CA	–	MIP	S1	–	–	–	–	–	BSA	1	[117]
C06	EC	CA	–	Antibody	N	–	SARS-CoV N	HCoV-NL63 N	FCoV 2 N	IFV	–	9	[118]
								HCoV-OC43 N					
							MERS-CoV N	HCoV-229E N		Sindbis			
								HCoV-HKU1 N					
C07	EC	CA	Magnetic nanobeads	ACE-2	S1	–	SARS-CoV S			VSV		4	[119]
							MERS-CoV S						
C08	EC	CA	–	Antibody	S1	–	–	–	–	Hemagglutin (HA)		1	[120]
D04	EC	POT	–	MIP	S1	–	MERS-CoV S	–	–	INF A (H1N1)	–	3	[124]
										INF A (H3N2)			
E01	EC	EIS	CuO_2_ nanocube	Antibody	S1	–	–	–	–	IFV A	–	2	[125]
										IFV B			
E02	EC	EIS	–	ACE-2	S1	–	–	–	–	–	IL-6	2	[126]
											Streptavidin		
E04	EC	EIS	Boron-doped diamond (BDD)	Antibody	S1	–	–	–	–	IFV B	–	1	[128]
E05	EC	EIS	–	ACE-2	S1			HCoV-OC43		INF A (H1N1)		7	[76]
										INF A (H3N2)			
								HCoV-229E		INF B (HSV2)			
										MHV			
										HSV-2			
E07	EC	EIS	Conducting nanocomposite	Engineered antibody	S(RBD)	–	–	–	–	–	IL-8	3	[130]
											IL 1B		
											TNF-a		
E08	EC	EIS	Au nanoparticles (AuNPs)	Aptamer	S1	–	SARS-CoV S	–	–	–	–	2	[131]
							MERS-CoV S						
E09	EC	EIS	Carbon nanodiamond	Aptamer	N	–	SARS-CoV S	–	–	Hemagglutin (HA)	–	2	[132]
E12	EC	EIS	–	ACE-2	S1	–	–	–	–	INF A (H1N1)	–	2	[134]
				CD-147 receptor						INF A (H3N2)			
E14	EC	EIS	Modified MWCNT + graphene	Antibody	S1	–	–	–	–	INF A (H1N1)		7	[136]
										INF A (H3N2)			
										INF A (H3N2)			
								HCoV-OC43		INF B			
								HCoV-229E		Avian Influenza A (H5N1)			
E16	EC	EIS	SiO2@UiO-66 nanocomposite	ACE-2	S1								[137]
E17	EC	EIS	carbon/graphene@PEDOT:PSS	Antibody	N			HCoV-NL63				1	[77]
E18	EC	EIS	Au nanoparticles (AuNPs)	Antibody	N	SARS-CoV-2 S(RBD)					IL 8	5	[138]
											IL 1B		
											TNF-a		
											IgG		
E19	EC	EIS	–	Aptamer	S1	–	–	HCoV-NL63	–	–	–	1	[139]
E20	EC	EIS	Zinc oxide reduced graphene oxide (bbZnO/rGO)	Antibody							IgG	1	[140]
E21	EC	EIS	Au nanoparticles (AuNPs)	Antibody	S1	–	SARS-CoV S	–	–	–	BSA	2	[141]
E22	EC	EIS	Gold nanostar	Antibody	N	–	MERS-CoV N	–	–	INF A	BSA	4	[142]
										INF B			
E23	EC	EIS	Carbon PEDOT:PSS Graphene	Antibody	N	–	–	–	–	INF A	BSA	4	[143]
										INF B			
E24	EC	EIS	–	Antibody	N	SARS-CoV-2 S	–	–	–	–	–	1	[144]
E25	EC	EIS	–	Aptamer	S(RBD)					INF A		5	[145]
E26	EC	EIS	Carbon nanodiamond	Antibody	S1	–	–	–	–	Hemagglutin (HA)	–	1	[146]
E27	EC	EIS	–	Synthetic peptide	S1	–	MERS-CoV S	–	–	–	B-1,4-GALT-5	2	[147]
E28	EC	EIS	Boron doped diamond (BDD)	Antibody	N	–	–	–	–	INF A	–	4	[148]
										INF B			
										RSV			
										EBV			
E29	EC	EIS	–	Antibody	S1	–	–	–	–	Hemagglutinin (HA)		1	[120]
E30	EC	EIS	MoS2- PDA nanosheet	Antibody	N	–	–	–	–	–	BSA	7	[149]
											Dopamine		
											Ascorbic acid		
											Hemoglobin		
											Transferrin		
											Human IgG		
											Rabbit IgG		
E31	EC	EIS	–	Aptamer	S(RBD)	–	MERS-CoV S	–	–	Hemagglutinin (HA)	–	2	[150]
F01	EC	OECT	–	Engineered antibody	S(RBD)	–	MERS-CoV S	–	–	–	GFP	2	[151]
					S1								
F02	EC	OECT	–	Antibody	S(RBD)	–	–	–	–	–	BSA	1	[152]
G04	EC	PEC	Au@TiO_2_	Engineered antibody	S1	–	–	–	–	–	Asuric acid	3	[156]
											l-lysine		
											l-glutamic acid		
G05	EC	PEC	Dioxide@bismuth tungstate (TiO_2_@Bi_2_WO_6_) nanocomposite	Antibody	N	–	–	–	–	–	CEA	4	[157]
											PSA		
											Insulin		
											Cardiac Troponin (cTnl)		
H02	EL	FET	Graphene	Antibody	S1	–	MERS-CoV S	–	–	–	–	1	[159]
				ACE-2									
H03	EL	FET	SWCNT	Antibody	S1	SARS-CoV-2 N	–	–	–	–	–	2	[43]
					N	SARS-CoV-2 S							
H04	EL	FET	MXenes/graphene	Antibody	S1	–	–	–	–	INF A (H1N1)	–	1	[160]
H05	EL	FET	TMCs	Antibody	S1	–	–	–	–	–	BSA	1	[161]
H08	EL	FET	Carbon nanotube (CNT)	Antibody	S1	–	SARS-CoV S	–	–	–	–	2	[164]
							MERS-CoV S						
H13	EL	FET	–	Antibody	N	SARS-CoV-2 S	SARS-CoV-2 N	–	–	INF A	–	4	[168]
										INF B			
H16	EL	FET	Reduced graphene oxide	Antibody	S1	–	–	–	–	–	BSA	1	[171]
H17	EL	FET	Graphene	Antibody	S(RBD)	–	SARS-CoV N	–	–	–	–	2	[172]
					N		MERS-CoV N						
H18	EL	FET	Graphene	Aptamer	S1	–	MERS-CoV S	HCoV-229E	–	–	BSA	3	[173]
H19	EL	FET	Graphene	Antibody	S1	–	MERS-CoV S	–	–	–	–	1	[174]
I02	EL	Tri-channel transistor	In_2_O_3_/ZnO	Antibody	S1	–	MERS-CoV S	–	–	–	–	1	[176]
I03	EL	Capacitive biosensor	Epitaxial graphene	Antibody	S1	–	–	HCoV-NL63	–	–	–	4	[177]
								HCoV-OC43					
								HCoV-229E					
								HCoV-HKU1					
I04	EL	Capacitive biosensor	–	ACE-2	S1	–	–	–	–	INF A (H1N1)	BSA	6	[178]
										PIV			
										Adenovirus			
										VZV			
										RyV			
I05	EL	Silicon nanowire array-based biosensor	Silicon nanowire (SiNWs)	ACE-2	S1	–	–	–	–	–	BSA	1	[179]
J01	OP	FL	Carbon nanotube (CNT)	Antibody	S(RBD)	–	SARS-CoV S	–	–	INF A	HSA	4	[62]
							MERS-CoV S						
J03	OP	FL	Carbon NANOTUBE (CNT)	–	S1	SARS-CoV-2 N	–	–	–	–	BSA	3	[182]
					N	SARS-CoV-2 S							
J04	OP	FL	Magnetic beads	Antibody	N	SARS-CoV-2 S	–	–	–	–	–	1	[183]
J05	OP	FL	Au@PtNPs	Antibody	N	SARS-CoV-2 S	–	–	–	H1N1 Hemagglutinin (HA)	–	6	[184]
										H1N1 Neuraminidase (NA)			
						SARS-CoV-2 S(RBD)				HBsAg			
										HCcAg			
J06	OP	FL	–	Aptamer	S1	–	MERS-CoV S	HCoV-NL63	–	–	–	5	[185]
								HCoV-OC43					
								HCoV-229E					
								HCoV-HKU1					
J07	OP	FL	Au nanoparticles (AuNPs)	Aptamer	N	SARS-CoV-2 S						7	[186]
K01	OP	CL	Co–Fe@Hemin	Antibody	S(RBD)	–	SARS-CoV S	HCoV-OC43	–	–	–	4	[187]
							MERS-CoV S	HCoV-HKU1					
K02	OP	CL	AuNPs	Antibody	N	–	SARS-CoV S	–	–	–	IgG	4	[188]
							SARS-CoV S(RBD)				IgM		
L01	OP	SPR	Graphene oxide	Aptamer	N	–	–	–	–	–	BSA	4	[189]
											l-Asparagine		
			Graphene oxide	Aptamer	N						l-Citrulline		
											Urea		
L07	OP	SPR	–	Engineered antibody	S(RBD)							1	[195]
M02	OP	LSPR	Silver nanotriangle array	ACE-2	S(RBD)	–	SARS-CoV S	HCoV-NL63	–	–	–	4	[197]
								HCoV-OC43					
								HCoV-229E					
M03	OP	LSPR	Au nanoparticles (AuNPs)	MIP	S1 (α)	–	–	HCoV-OC43	–	–	–	3	[198]
					S1 (β)			HCoV-229E					
					S1 (γ)			HCoV-HKU1					
N01	OP	Fiber optics (SPR)	–	MIP	S1	–	MERS-CoV S	–	–	–	–	1	[199]
N02	OP	Fiber optics (SPR)	–	Aptamer	S (S1 + S2)	–	MERS-CoV S	–	–	INF A (H1N1)	BSA	4	[200]
										Hemagglutin (HA)			
N03	OP	Fiber optics (SPR)	–	Peptide	Protease	–	–	–	–	–	Albumin	4	[63]
											Thrombin		
											Papain		
											Denatured protease		
N04	OP	Fiber optics	Au nanoparticles (AuNPs)	Antibody	S1	–	MERS-CoV S	–	–	–	–	1	[201]
N05	OP	Fiber optics (FL)	Polystyrene microspheres	Antibody	N	–	–	HCoV-NL63	–	–	–	4	[202]
								HCoV-OC43					
								HCoV-229E					
								HCoV-HKU1					
N06	OP	Fiber optics (BLI)	–	Antibody	S1	–	SARS-CoV S	–	–	–	–	3	[203]
					S(RBD)		MERS-CoV S						
							SARS-CoV N						
O02	OP	SERS	Au nanoparticles (AuNPs)	Antibody	S1	–	–	–	–	HIV p24	CEA	5	[204]
											AFP		
										EPV GP350			
											BSA		
O05	OP	SERS	Au nanoparticles (AuNPs)	Antibody	S1		SARS-CoV S					2	[207]
							MERS-CoV S						
O08	OP	SERS	Au nanoparticles (AuNPs)	Antibody	N	–	–	–	–	INF A (H1N1)	–	4	[210]
										INF A (H3N2)			
										INF A (H5N2)			
										INF B			
O10	OP	SERS	Magnetic nanoparticles	Engineered antibody	S1	–	–	HCoV-HKU1	–	–	–	1	[212]
O12	OP	SERS	Core–shell Au nanoparticles (AuNPs)	Antibody	S(RBD)	–	SARS-CoV S	–	–	–	–	2	[214]
							MERS-CoV S						
P01	OP	Colorimetric	Au nanoparticles (AuNPs)	ACE-2	S1	–	–	–	–	INF A (H1N1)	–	4	[215]
										INF B			
										HSV-2			
										MHV			
P02	OP	Colorimetric	Core–shell Au@Pt nanoparticles	Antibody	S1	SARS-CoV-2 N	–	–	–	–	GOX	6	[216]
											Collagen		
											BSA		
											Amylase		
											Lysozyme		
P03	OP	Colorimetric	Core–shell Au@Pt nanoparticles	Antibody	N	–	SARS-CoV N	HCoV-NL63	–	INF A (H1N1)	Bacterium (*Staphylococcus aureus*, *Pseudomonas aeruginosa*)	16	[217]
										INF A (H3N2)			
								HCoV-OC43		INF B (Victoria)			
										INF B (Yamagata)			
							MERS-CoV N	HCoV-229E		MeV			
										MuV			
								HCoV-HKU1		RuV			
										VZV			
Q02	OP	Phononic sensors	Graphene	Antibody	S(RBD)	–	MERS-CoV S	–	–	–	–	1	[219]
R01	ME	Micro-cantilever	–	Antibody	S(RBD)	–	MERS-CoV S	–	–	INF A	–	2	[220]
					N								
T01	TH	Thermal assay	–	MIP	S(RBD)	–	–	–	–	–	HSA	3	[222]
											ORF8		
											IL-6		
U04	CO	SERS (+ LFA)	Au nanoparticles (AuNPs)	Antibody	S(RBD)		MERS-CoV S			INF A	Bacterium (*Pneumoniae*)	3	[226]
U06	CO	SERS (+ LFA)	Au nanoparticles (AuNPs)	ACE-2	S(RBD)	–	SARS-CoV S	–	–	–	BSA	4	[228]
							MERS-CoV S				HSA		

^a^*INF* Influenza, *AIV* Avian Influenza Virus, *HIV* Human Immunodeficiency Virus, *HPV* Human Papillomavirus, *RSV* Respiratory Syncytial Virus, *EBV* Epstein-Barr Virus, *HRV* Human Rhinovirus, *MHV* Mouse Hepatitis Virus, *NGAL* Neutrophil Gelatinase-Associated Lipocalin (Puumala virus) , *RuV* Rubella virus, *MuV* Mumps Virus, *MeV* Measles Virus, *EBOV* Ebola Virus, *VSV* Vesicular Stomatitis Virus, *VZV* Varicella-zoster Virus, *PIV* Parainfluenza Virus, *HBsAg* Hepatitis B Surface Antigen, *HCcAg* Hepatitis C Virus Core Protein, *HCV* Hepatitis C

^b^*BSA* Bovine Serum Albumin, *HSA* Human Serum Albumin, *IL-6* Interleukin 6, *IL-8* Interleukin 8, *GFP* Green Fluorescent Protein

Clinical specificity can be obtained by comparison with RT-PCR results. As we mentioned in the previous section, 14.6% (23/158) of the articles presented a clinical validation (Table 3). The results were less varied compared to that of clinical sensitivity. Unless the sample size of PCR-negatives was small (≤ 10), the clinical specificity of the reported nanobiosensors was over 86.0%, and 12 studies reported that there were no false-positive results in at least one experimental design.

#### User-friendly tests (U)

The development of an easy-to-use test is crucial during a global pandemic. If a test is easy for individuals to conduct for themselves or others with the proper instructions, the enormous burden of testing capacity would be relieved. In that case, the healthcare system can efficiently allocate the limited testing resources for molecular tests (i.e., RT-PCR) to the areas in urgent need. In many countries, this strategy somewhat worked with the help of RATs at the peak point of the last wave caused by the Omicron variants.

Unfortunately, most nanobiosensors are difficult to handle for individuals at home. There are three hurdles in this criterion: (a) the whole test process, from the sampling to outcome, needs to be simplified to a few steps since complexity of the procedures usually lowers the possibility of a successful test; (b) the sampling also needs to be conducted by the individuals, so nasopharyngeal (NP) or oropharyngeal (OP) swabs are inappropriate for self-collection; (c) if the assistance of instruments is required for the result confirmation, they can be handled with ease under the instructions. Numerous studies claimed a simple procedure, but this criterion might be the hardest to evaluate because there is no quantitative standard.

One persuasive approach to address this issue is the adoption of pre-existing commercial products, such as glucometers and pregnancy tests. These two cases are not only rare successful examples of biosensors in history but also current indicators of the accessibility of previously difficult tests for ordinary people. For example, the glucose strip is produced and sold on a large scale due to the high demand for this routine test. So, the facilities to manufacture the glucose strip are already established, and they can produce several million sensors per day [126]. Kiew et al. (E02) suggested glucose strip-inspired sensors for the detection of SARS-CoV-2 [127]. Although this article mainly focused on the merit of mass production to match the testing demand, their suggestion offers a way forward. Singh and Ray et al. suggested an innovative way to use an off-the-shelf glucometer with customized electrodes [229].

Regarding self-collection, the nanobiosensor tests need to consider the sampling of easy-to-collect specimens instead of NP and OP. For example, saliva is a rational specimen considering that the salivary droplet is the most critical source of human-to-human transmission of SARS-CoV-2 [230] and can be obtained in a non-invasive manner, thus meeting the requirements for self-collection and enabling routine self-testing. However, the value of saliva as a test sample is still controversial because there can be a huge difference in the contents and concentration among sampling methods (mouthwash, oral swabbing, coughing, drooling). In addition, the quality of the sample obtained from the same individual is not always the same due to anomalous effects, including viscosity, mucous contents, and potential inhibition from food particles. Therefore, it is hard to guarantee that the best performance of the test can be drawn from self-collected samples. A total of 14 studies examined the sensing performance under saliva-related environments, including real, diluted, filtered, or artificial saliva. Among them, tweilve studies compared the results with the sensing performance under a buffer environment (A02, A07, B13, C08, E05, E29, F01, H13, H16, O02, Q02, U06). These studies found that the LoD was lowered when the sample matrix changed from the ideal buffer to a complex environment. The difference in the LoD between the buffer and saliva was one or two orders and could be a bottleneck with the relatively low amount of target antigen in saliva.

#### Rapid and robust tests (R)

Aside from sensitive and specific detection, the most frequently mentioned claim for nanobiosensors is rapid detection, indicating that the sensor outputs the results with a fast response. Under the spreading of infectious viruses, rapidity is more important than ever before. Because the number of infected individuals increases by hundreds of thousands every day, the overwhelmed testing capacity cannot be relieved by a method requiring a long-term process. For instance, RT-PCR is usually a four- to six-hour process, implying that the realistic possibility of handling all the cases that need to be tested is not likely with the current strategies. Preferably, the test time should be shortened to a similar level to that of RATs (15 to 30 min). Understandably, the quicker, the better, unless other sensing performances are lost.

Rapidity is complicated when we discuss nanobiosensors. Although the usual response time of most transducers is theoretically in the range of sub-seconds, the additional signal processing time often varies with the detection methods [231]. In addition, there is conceptual confusion in defining the standard for rapid detection since every study has different definitions of rapidity, including detection time, response time, and total turn-around time. The detection time is the required time for obtaining detection results from the specimen, while response time refers to the exact time interval between the stimuli engagement and the signal generation. Because the head-to-head comparison among these studies is illogical, we marked all the articles that claimed rapid detection in Table 5. However, specific information on rapidity is only mentioned when it refers to the exact sample-to-result time. The results show that 83.5% (132/158) of studies claimed rapid detection, and one-third offer a sample-to-result time of under 30 min.

**Table 5:** The ASSURED-related claims and MORE-related issues in nanobiosensor research

ID	ASSURE-related claims								MORE-related Issues								Note
	Platform		Sample preparation		Sensing performance		Measurement		Confrontation ability to evolving situation				Smart features	Global distribution			
	Low-cost	Disposable	Self-collection	Low volume	Rapid	Reproducible	Non-buffer environment	Portable equipment	Multiple antigen Detection	Antibody detection	Variant	Other viruses	Data transmission	Mass testing	Stability	Reusability	
A01					●												[82]
A02					● 30 min		● Untreated Saliva	● Portable Potentiostat	● S1 + N								[83]
A03					●		● Lysis Buffer	● Portable Potentiostat	● S1 + N								[84]
A04						●									●		[85]
A05			●		●			● Portable Potentiostat					● Bluetooth				[86]
A06	●				● [Saliva] 5 min [OP] 35 min		● Saliva								● 42 Days	● Twice More	[87]
A07	●				●		● Saliva	● Portable Potentiostat							● 4 Weeks		[88]
A08	●					●		●							● 30 Days		[89]
A09		●				●	● Diluted Saliva								● 5 Days		[90]
A10						●									● 35 Days		[91]
A11					● 30 min	●		●				● INF A (H1N1)			● 7 Weeks (at 25℃)	● 30 Times	[92]
A12					● 2 h	●									● 10 Days		[93]
A13					●			●									[94]
A14					●										● 21 Days (at 4℃)	● 5 Times	[95]
A15	●				●			● Portable Potentiostat					● WiFi				[96]
A16					●			● Portable Potentiostat			● Alpha Beta Delta		● Wireless				[97]
A17	●				●												[98]
A18	●				●												[99]
A19	●				●	●									●	●	[96]
B01	●				●	●											[101]
B02					● 45 min			● Portable Potentiostat									[102]
B03	●						● Diluted Saliva	● Miniaturized EC cell									[75]
B04	●				●	●									● 10 Days (at 4℃)		[103]
B05	●				● 35 min	●									● 30 Days		[104]
B06	●				● 6.5 min	●		●			● Beta				● 7 Days (at 4℃)		[105]
B07					●												[106]
B08	●				●	●											[107]
B09	●				● 15 min	●		● Portable Potentiostat			●						[108]
B10					● 30 min										● 21 Days		[109]
B11	●				● 45 min						● Omicron						[110]
B12	●				●	●		● Hand-held Portable Potentiostat							●	● 9 Times	[111]
B13		●			●	●		● Portable Potentiostat							● 15 Days		[112] 12/9/2023 1:19:00 PM
C01	●				●												[113]
C02					●										● 9 Months (at 4℃)		[114]
C03	●	●			●							● INF A (H1N1)					[115]
C04				●	● < 60 min		● Serum and Diluted Serum	● Portable Potentiostat					● Smartphone				[116]
C05																	[117]
C06	●			● 20 uL	● 70 min			● Portable Potentiostat			● Alpha Beta						[118]
C07	●				●	●		●					● Smartphone		● 20 Days (at 4℃)		[119]
C08		●		●	●	●		● Portable Potentiostat							● 3 Days		
D01					●												[121]
D04					●	●	● Saliva										[124]
E01	●	●			● 20 min	●									● 14 Days		[125]
E02	●		●		●									●			[126]
E03				● 1 uL	● 21 min												[127]
E04					●												[128]
E05	●	●		●	● 4 min	●	● VTM and Saliva	● Handheld Potentiostat			● Alpha		● Smartphone		● 10 Days (at 25℃)		[76]
E06	●		●		●												[129]
E07	●				●	●									● 7 Weeks (at 4℃)	● 5 Times	[130]
E08	●				●										● 21 Days		[131]
E09	●				●	●	● Diluted Saliva								● 10 Days	●	[132]
E10	●					●									● 4 Weeks		[7]
E11	●				● 15 min	●		● Portable Potentiostat									[133]
E12				●	●	●					● Alpha						[134]
E13	●																[135]
E14	●				●												[136]
E15	●	●			●												[74]
E16	●				●	●										● 10 Times	[137]
E17	●				● 30 min	●		● Portable Bi-Potentiostat			● Alpha		● Wireless		●		[77]
E18															● 6 Weeks	● 4 Times	[138]
E19					● 15 min	●					● Alpha				● 30 Days		[139]
E20	●	●		● 10 uL	● 35 min	●		●									[140]
E21					●						● Alpha Beta Delta						[141]
E22		●			● 40 min	●		● Portable Electrical Readers									[142]
E23	●				● 10 min	●		● Portable Bi-Potentiostat			●		● WiFi				[143]
E24	●															● > 30 Times	[144]
E25	●				●	●									● 14 Days		
E26	●				●	●									● 3 Weeks	●	[145, 146]
E27	●				●	●		● Portable Potentiostat							● 20 Days		[147]
E28					●	●											[148]
E29	●	●			●	●											[120]
E30	●				●												[149]
E31	●				●			●			● Alpha Delta						[150]
F01		●		● 5 uL	● 15 min		● Saliva	● Hand-held Reader									[151]
F02										● Anti-S1 Antibody							[152]
G01	●				●												[153]
G02	●					●											[154]
G03	●					●									● 3 Weeks		[155]
G04	●				● 30 min	●									● 21 Days		[156]
G05						●									●		[154]
H01																	[158]
H02					●		● Diluted VTM										[159]
H03	●				●				● S1 + N								[43]
H04	●				●							● INF A (H1N1)					[160]
H05					●												[161]
H06	●				● 20 min					● Anti-S1 Antibody							[162]
H08	●				●	●										● 5 Times	[164]
H09									● S1 + N								[165]
H10	●				●	●		● Portable Integrated Platform							● 228 Hours (at 4℃)		[73]
H11					● 20 min			● Portable FET Sensor			● Delta Plus Kappa						[166]
H12					● 20 min												[167]
H13		●			● 30 min		● Artificial Saliva	● Portable Reader					● Bluetooth				[168]
H14					●	●		● Portable Reader					● Bluetooth				[169]
H15					●												[170]
H16					●		● Saliva-relevant Fluids										[171]
H17					● 20 min			● Portable Reader	● S1 + N		● N501Y, D614G, Y453F, Omicron-B1.1.529		● Wireless				[172]
H18	●					●		● Home-built Portable Readout Electronic Unit		● Anti-S1 Antibody					●		[173]
H19	●				●										● 6 Weeks		[174]
I01	●				●	●									● 10 Days	● 2 Times	[175]
I02					●												[176]
I03	●			●	●			● Biopotentiostat			● Alpha					● Multiple Times	[177]
I04					●	●									● 7 Days (at 4℃)		[178]
I05	●				● 1 h												[179]
I06	●				●							● INF A					[180]
J01					●												[62]
J02	●			●	●				● S1 + N								[181]
J03				●	●				● S1 + N						● 6 Days		[182]
J04																●	[183]
J05	●	●			● 25 min			●			●		● Bluetooth		● 15 Days		[184]
J06																	[185]
J07																	[186]
K01	●				● 16 min			●					●				[187]
K02	●				●	●									● 30 Days		[188]
L01					●												[189]
L02					●												[190]
L03					● 75 min												[191]
L04					●												[192]
L05					●												[193]
L06					●	●									● 21 Days	● 6 Times	[194]
L07					●			● Portable SPR			● Alpha Beta Delta Omicron						[195]
M01					●												[196]
M02	●				● 20 min			● Portable UV–Vis spectrophotometer				● CoV-NL63					[197]
M03	●	●			● 30 min		● Diluted Serum	● Portable Reader			● Alpha Beta Gamma				● 30 Days		[198]
N01	●				●												[199]
N02	●				●												[200]
N03	●						● Serum										[63]
N04	●				●			●							● 14 Days	● Once More	[201]
N05	●			●	●			● Portable Microplate Readers					● Smartphone				[63]
N06					●										● 14 Days		[201]
O01				●	●												[69]
O02						●	● Saliva, Serum, and Blood								● 15 Days		[204]
O03	●								● S1 + N								[205]
O04	●				●	●											[206]
O05					●												[207]
O06					●												[208]
O07					● 15 min		● Mixed Protein Solution, Urine, Blood	● Portable Raman Spectrometer									[209]
O08					● 30 min			● Portable Device									[210]
O09					●		● Cell Lysate										[211]
O10					● 30 min			● Handheld, Battery-Operated Device			● Alpha Beta Delta	● HKU1					[212]
O11					●												[213]
O12											● N501Y E484K T478K						[214]
P01	●				●	●							● Smartphone		● 7 Days (at 4℃) 4 Days (at RT)		[215]
P02					●												[216]
P03	●														● 35 Days (at 4℃, RT, 37℃)		[217]
Q01					● 50 min												[218]
Q02					●		● Artificial Saliva										[219]
R01	●				●						● Alpha				● 15 Days		[220]
S01	●				●			● Portable MPS Systems									[221]
T01	●	●		●	● ~ 15 min						● Alpha				●		[222]
U01	●	●	●	●	●	●				●					● 14 Days		[223]
U02					●												[224]
U03	●	●			●	●									● 4 Weeks (at 4℃)		[225]
U04					● 20 min			● Portable Raman Spectrometer									[226]
U05	●				●												[227]
U06	●			●													[228]

Robustness is an important criterion as well and indicates how long the functionality of the test is maintained without any special conditions [232]. Ideally, the test performance should not be dependent on temperature and humidity. This criterion highlights the advantage of antigen-based tests because nucleic acids are highly unstable and easily denatured. Though it is difficult to evaluate exactly during research, the shelf-life can be used to assess robustness. The results show that 54 of 158 studies (34.2%) claimed robustness, and among them, twelve studies verified that the stability of the test results was preserved for more than 30 days at room temperature (A07, A10, A11, B05, C02, E07, E10, E18, H19, K02, M03, P03). The longest shelf-life was seven weeks, with a signal decrease of 1.27% at room temperature (A11). Similarly, another study reported a shelf-life of 42 days at room temperature with a signal decrease of 8.5% (A06). Interestingly, they also tested the sensor stability at 37 °C and reported a signal decrease of 11.9%. Several studies reported stability at 4 °C (A14, A17, B04, B06, C02, C07, E07, H10, I04, P01, P03, U03), and the longest period was nine months (C02).

#### Equipment-free tests (E)

Equipment is a fundamental dilemma in test development. High-performance and expensive equipment have a better possibility of obtaining a better result. Nevertheless, the equipment-free test is one of the ultimate goals of diagnostic tests that can solve many diagnostic issues. Since a lack of testing capacity worsens the situation during pandemics caused by infectious viruses, equipment-free tests would be helpful to slow the virus spreading with more frequent and repetitive testing.

A large portion of nanobiosensors is highly dependent on equipment; however, portable equipment may address this issue. Portable equipment is affordable and does not take up much room. The transport cost is not relatively high because portable equipment can be the size of a hand-held device and can be shipped in small boxes. This means portable equipment can be used for at-home testing or to set up temporary testing sites at a low cost. However, there is a gap between portable and bench-top equipment in terms of specification, performance, and functionality. The issue is if portable equipment can meet the minimum requirements for detecting viral proteins. To answer this question, we compared references with identical principles: S protein detection in a buffer using voltammetric detection. In this category consisting of 14 studies, seven operated a bench-top EC workstation, and seven ran a portable device. The LoD distribution of the former group was slightly better than that of the latter group. It is hard to compare these studies because each study had its own unique design and different conditions, but higher analytical sensitivity might be derived from the equipment spec. However, the difference in the averages was not significant, and several studies using portable devices showed results in an acceptable range.

#### Deliverable tests (D)

The last criterion of ASSURED is the ability of tests to be deliverable to the end-users. Deliverability indicates the accessibility of the technology to the public and is closely related to the requirements for the final stage of the test at the testing site. The testing sites here varied from at home to public spaces, including schools, workplaces, hospitals, train stations, and airports. Furthermore, there is a need to set up temporary testing sites within a very short time. There are some postulations to be addressed in transferring the test to these testing sites. For example, some methods need reagents that require cooling and refrigeration facilities at the testing site. If the test requires cooling or refrigeration, the flexibility would be degraded, and the distribution of the test would be limited. Specialized storage is also problematic regarding at-home testing since the test reagent may not be stored in household refrigerators. For these reasons, deliverability is closely related to the storability at room temperature of the test in the rigidness criteria.

### MORE issues

Although the ASSURED criteria are important guidelines focusing on infectious diseases for over a decade, they cannot completely cover present issues during the COVID-19 pandemic. Most importantly, SARS-CoV-2 rapidly spread all over the world before proper actions were taken, demonstrating how different this virus is compared to other infectious diseases having relatively low reproductive numbers. A large portion of infected individuals causes unexpected variables in the process of virus containment, and the potential long-term effects of SARS-CoV-2 cause other serious consequences. Therefore, we believe more criteria should be established to address these additional issues aside from the existing ASSURED criteria. We chose newly emerging issues for diagnostic tools and arranged them into four categories, multiplexed detection (M), on-circulating variant (O), real-time connectivity (R), equity for global health (E), (MORE). The related articles are presented in Table 5.

#### Multiplexed detection (M)

Even though the vaccine and therapeutics far lowered the risk of COVID-19, SARS-CoV-2 will continue to coexist with us for a while. Even in the early stages of the pandemic, some experts predicted the transition to the endemic stage [233]. In other words, SARS-CoV-2, which keeps mutating, will circulate through the world with other seasonal diseases (e.g., influenza). This prediction implies the possibility of co-infection with other viruses or bacteria [234, 235]. In fact, we have realized through the last two winters that the infection rate of COVID-19 worsens during flu season. This concern was termed a “twindemic” since there is evidence that the infectivity of SARS-CoV-2 can be affected by other viruses [236]. In that case, the sole detection of SARS-CoV-2 would not be important in the future. Therefore, several studies have already suggested multiplexing techniques to detect multiple viruses along with SARS-CoV-2 at the same time.

As seen in Table 4, 35 studies included a test with influenza antigens. Those tests were designed to assess the specificity or selectivity of the sensor against real targets, antigens of SARS-CoV-2, and its variants. Since the early symptoms of COVID-19 are very similar to the seasonal flu, the discrimination between these two viruses is important. In this context, several studies investigated the multiplexed detection of SARS-CoV-2 and influenza (A11, C03, H04, I06). Nanobiosensors are an ideal platform for multiplexed immunoassays due to their enhanced sensitivity, reproducibility, and reliability. Also, nanobiosensors offer less sample consumption and a relatively short test time.

Moreover, the multiplex concept is also useful in the combination of antigen and antibody detection, especially in biofluids like serum. As mentioned above, the antibody level reflects both a past infection and the present status of the immunity, so multiplexing can offer a wider time frame for diagnosis [237]. In addition, multiplexing is helpful in monitoring vaccine efficiency and seroprevalence assessments of immunity in a population [29]. Six studies suggested the detection of both antigens and antibodies (A13, F02, H06, H18, J02, U01). The LoD of those three platforms for IgG or IgM was 1 fg/mL, 0.36 fg/mL, and approximately 0.3 pg/mL, respectively. These results are impressive considering the usual LoD of common serological tests. However, these cases must be interpreted with caution because both antigen and antibody were not detected on a single platform. Instead, two parallel platforms were used by exchanging biorecognition elements. Therefore, other nanobiosensors can also be converted to the antigen-targeted concept without additional effort.

#### On-circulating variant (O)

The threat of SARS-CoV-2 variants may cause COVID-19 to change from the one that had spread at the very early stage [238]. The mutation of the virus is a normal process to adapt to natural defenses. RNA viruses have an especially high mutation rate, so mutations could happen all the time. However, the consequences were fatal in the case of SARS-CoV-2 because ultra-fast, population-scale, and globe-wide spreading gave the virus innumerable opportunities for evolution. Although most mutations are expected to be either neutral or middle deleterious, some lead to different characteristics in pathogenicity, infectivity, transmissibility, and antigenicity [239, 240]. This means the variants are generally more transmissible than the wild-type or have the ability to evade a vaccine. The statistics show that every new variant drove the start of new waves of infection.

For this reason, the WHO carefully monitors the newly identified mutations and designates them as variants of concern (VOCs) and variants of interest (VOIs) based on their degree of global health significance. So far, there have been five major variants of SARS-CoV-2, including B.1.1.7 (Alpha), B1.351 (Beta), P.1 (Gamma), B.1.617.2 (Delta), and B.1.1.529 (Omicron). Broadly, these variants could be a serious problem on two counts. First, major mutations usually occur at the RBD of the S protein. Since RBD plays a role in the host cell entry, its mutation directly affects viral transmissibility via interactions with ACE-2 [241, 242]. Until now, most VOCs have had more infectious characteristics. For example, Alpha was estimated to be 50% more transmissible than wild-type viruses [243]. Delta has approximately three-fold greater infectivity than other variants [244]. More recently, Omicron, which contains mutations and has three-fold greater infectivity than Delta, showed more aggressive spreading, as well as Omicron variants and sub-variants. Omicron spreading was significantly faster than Delta variants, and the reproductive number was around seven [245, 246]. Second, there is a possibility of vaccine evading. For instance, though Beta and Gamma are not more transmissible than Alpha, they lowered the effectiveness of neutralizing antibodies, drugs, and vaccines [10].

In the development of diagnostic tests, mutations are problematic because the variants can affect the performance of the tests. Supposing that the sensitivity of the tests is reduced every few months, their value for practical use also keeps changing. The mutation in the S protein, especially in RBD, increases transmissibility. This fact implies that the biorecognition elements for S or S(RBD) might be venerable to a variant issue. Only one study (I03) conducted an antibody-based detection of Alpha variants as a practical demonstration in diluted saliva [177]; the signal difference between Alpha and wild-type was not provided. Another study (O10) using an engineered antibody reported that the signal differences among Alpha, Beta, and Gamma were small within a margin of error [212]. Two MIP-based studies (B09, M03) also showed a small difference among Alpha, Beta, and Gamma from the originally imprinted wild-type antigen, though the results were clearly distinguishable from the background or non-target viral proteins [108, 198]. Moreover, one ACE-2-based study (B06) showed interesting results [105]. In this very first report for variant detection, this study obtained an even higher response with Beta variants and also validated the test using clinical samples. Because the mutation of S(RBD) increases the affinity against ACE-2, the sensitivity of the sensor was enhanced against variants. N protein is often considered relatively free from variant issues compared to S protein. One study (C06) showed that the signal in Alpha and Beta was higher than that in the wild-type [118]. Another study (E23) demonstrated signal differences with 22 clinical samples obtained from patients diagnosed with the Alpha variant [143].

#### Real-time connectivity (R)

The fast-spreading rate of SARS-CoV-2 and a large portion of asymptomatic patients make containing the virus very difficult. It has so far been shown that quarantines, travel restrictions, and social distancing are the most effective strategies for controlling this sort of highly infectious disease. To conduct this plan of action, aggressive contact tracing, the confirmation of the infection, and the recovery need to be simultaneously monitored by the centralized healthcare system. So, test results should be shared with the authorities, and the proper measures and actions can proceed. The use of digital technologies to identify both active and recovered infected cases has also been implemented successfully to manage the spread of COVID-19 [247]. To do so, one crucial requirement for nanobiosensors is real-time connectivity. Fortunately, the integration of this function is not difficult in the world of smartphones. The integration of a module for wireless connection to the nanobiosensor is now achievable in an inexpensive manner.

In this context, several studies describe the real-time connectivity of their developed nanobiosensors. First, there are customized devices completely designed by authors (A05, A15, E17, H13). Beduk et al. (A05) developed a handmade POC potentiostat connected to a smartphone with a custom mobile application [86]. This device, which has built-in memory, supports both Bluetooth and USB-C connectors to sync to a smartphone. They did not, however, present details for the operation of the custom-made mobile application. Fortunati et al. (A15) developed a smart potentiostat that uses Wi-Fi to connect to a cloud environment directly. This device does not require external devices like PCs, tablets, or smartphones [96]. Salahandish et al. (E17) developed a customized dual electrochemical biosensor that enables binary electrochemical data acquisition (Bi-ECDAQ) and Bluetooth [77]. A few months later, they also developed a custom-designed dual-working electrode immuno-biosensor (BiSense), which has built-in Wi-Fi connectivity. P.-H. Chen and C.-C. Huang (H13) proposed an electrical double layer (EDL)-gated FET biosensor [168].

Second, there are studies that integrated their invention into the electrodes used in commercial equipment, including potentiostats (C04, E05), portable FET measurement stations (H14), photometers (J05), and glucometers. Currently, most commercial portable devices support wireless protocols like Bluetooth, near-field communication (NFC), and Wi-Fi. In addition, the recent products available on the market usually present software development kits for mobile applications. Among them, two studies (C04, E05) showed that their sensor electrodes could be connected to various types of potentiostats. Since these devices were running with both PC-based bench-top equipment and smartphone-based portable equipment, the measurement can be conducted according to the circumstance.

#### Equity for global health (E)

The current COVID-19 pandemic surpasses the advances in modern globalization in the twenty-first century [248]. As we realized for the last three years, this highly infectious virus cannot be contained by the sole efforts of a single community, society, or nation. Nowadays, our worlds are closely connected on every level and thus affected interactively. This means crucial responses to COVID-19 need to move forward under global-level cooperation [249]. However, joint efforts among nations still seem very idealistic when considering the international situation. There are several different levels of inequity to consider. First, huge gaps in the fundamental health system and public hygiene exist between advanced and developing countries; infectious diseases cause more dangerous aftermath in low-income countries and communities [9]. Second, there is a staggering inequity in the aspect of diagnostic infrastructure. The current diagnostic methods, like RT-PCR, have intensified in high-income countries. Another inequity to consider is vaccine distribution [250]. Regarding the emergence of the new variants, especially, different levels of vaccination rates have caused other viral mutations. For these reasons, the alternative methods should cover the area that RT-PCR cannot reach.

Nanobiosensors, as an alternative tool, should cover the area that RT-PCR cannot reach. This issue is closely intertwined with the criteria mentioned above, including the affordability, user-friendliness, rapidity, rigidity, and deliverability of tests. In terms of testing capacity, the tests need to meet the requirement for mass testing and stability. Mass-producible platforms can contribute to the global-scale distribution. Stability is also important during transport to isolated areas or undeveloped countries. Interestingly, 18 studies have demonstrated the reusability of their tests (A06, A11, A14, A17, A19, B12, E07, E09, E16, E18, E24, E26, H08, I01, I03, J04, L06, N04), where four claimed that the performance loss was in an acceptable range even after more than ten uses (A11, A19, E16, E24). Reusing the test is useful in resource-limited circumstances to expand testing capacity.

## Conclusion and future perspectives

In this review, we first summarized the three-year history of the COVID-19 pandemic and the advantages and limitations of the current diagnostic toolbox. During this period, scientific research for COVID-19 had been published at a record pace, including hundreds of concepts for nanobiosensors for detecting SARS-CoV-2 through targeting nucleic acids, antibodies, and antigens of the virus. Among them, about 158 studies particularly claimed the rapid and sensitive detection of SARS-CoV-2 antigens, thus also achieving the direct detection of the virus itself at the early stage of the infection. Considering the relatively short circulating period of the newly emerged virus, the flourishing of published articles is unprecedented in the entire biosensor history, implying the urgent importance of this subject. Thereafter, we focused on the analytical performance of those reported nanobiosensor studies and the contribution of the nanomaterials. We then discussed the diagnostic requirements for a pandemic using the WHO’s ASSURED criteria and the new MORE virus-specific issues.

**Fig. 5:**
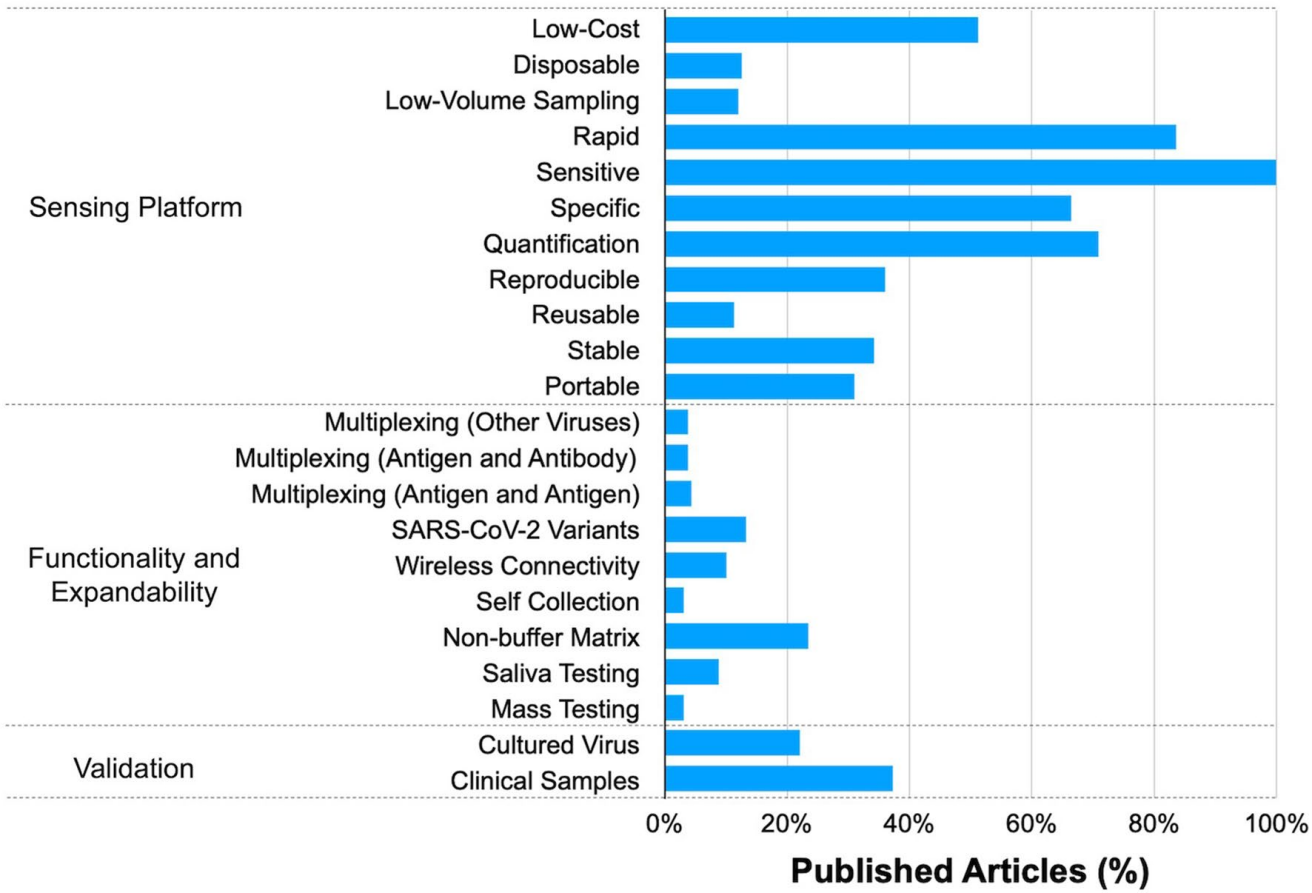
The analysis based on key claims and approaches of the relevant studies. (bar graph: a fraction of the relevant cases among a total of 158 published articles)

In Fig. 5, we illustrated how these issues have been addressed in the previous articles based on the key claims of the article. Considering that all the studies directly or indirectly emphasize the sensitive detection of SARS-CoV-2, we can estimate the relative weight of other issues in counteracting infectious diseases. It tells us about the necessary direction for nanobiosensor research in the future and also shows both positive and negative aspects of its current status. The major strength of nanobiosensors stems from excellent analytical sensitivity and quantification ability compared to other antigen-based detection methods, such as conventional in vitro diagnostics. On the other hand, the critical weakness of nanobiosensors is a lack of standardization. This includes low selectivity, performance decrement in real samples, and the limited reproducibility of results. Most studies in the scientific literature are dependent on lab-specific protocols and conducted under different measurement conditions, which makes the parallel comparison difficult. These would be essential assignments in the future to allocate the role to nanobiosensors in urgent circumstances. In this context, we need to give attention to conventional immunoassays like ELISA as a standardized model of newly developed technologies. It is also the reason why a large amount of nanobiosensor research provides performance comparisons with ELISA tests under identical conditions.

It is worth noting that the studies with clinical samples are preferably referred for future development. Cross-validation with the gold standard RT-PCR is especially valuable information. In addition, ASSURED and MORE criteria illustrate the eventual objective of all kinds of nanobiosensors. The issues we handled in each section have great implications for the future development of nanobiosensors. Therefore, the studies satisfy these criteria as much as are worth noticing. In this context, the studies that suggested the prototype or adoption of the existing commercial platform provide important points in terms of the feasibility of the concept. An additional point in future development is the integration with smartphone-based platforms, along with artificial intelligence-assisted and cloud-connected data analysis. The smartphone, which has the potential to be a highly personalized diagnostic device, can contribute to data acquisition, processing, storage, and sharing. In the case of infectious disease, this approach is particularly important because it provides real-time monitoring and reactive counteraction to the spreading of the virus with accurate data extraction in terms of both quality and quantity.

Although the battle against COVID-19 is now entering the final stage as the risk of the virus continuously decreases, its management is still important for the following reasons. First, the virus will not simply disappear. Instead, SARS-CoV-2 will coexist with us for a long time, like the flu. Second, we still do not know the exact long-term consequences of COVID-19, the so-called “Long COVID.” There have been several reports that patients after recovery have sequelae, including cardiovascular, pulmonary, neurologic, mental, and emotional disorders [251, 252]. In fact, the sequelae are somewhat common to some other viral infections (e.g., hepatitis B), and the long-term consequences were also observed in survivors of previous SARS-CoV [253, 254]. These long-term effects may become an even larger problem in the case of a mass-spreader like SARS-CoV-2. Third, COVID-19 is still dangerous for some population groups, including the older age group with underlying diseases. Despite the unprecedented scale of vaccination and the recent dominance of variants that are more infectious but less dangerous, the mortality in this subpopulation is exceptionally high. So, to protect the entire community, the continuous effort to control COVID-19 is unavoidable.

It is evident that COVID-19 is not the last pandemic in human history. Many experts have warned that outbreaks of other infectious diseases will be more frequent in the future. In 2022, we observed the unexpected spreading of once-dismissed viruses, including monkeypox and polio. Monkeypox had previously never spread outside central and west Africa. Polio, a virus feared in the middle of the twentieth century, had not been detected in large-scale populations. Both monkeypox and polio have since spread on a global scale similar to SARS-CoV-2 and its variants. It is worth noting that the development of transportation allows the world to act as a single society, and overpopulation and over-urbanization offer a perfect opportunity for viruses to travel, proliferate, and mutate at a remarkable speed. Because the fertile environment for the virus is already set, we cannot be sure the consequence of the next virus will differ from SARS-CoV-2. One important lesson in this pandemic is the awareness of the necessary need to prepare various alternative or complementary options to support RT-PCR-based diagnostic testing through global-scale testing to keep up with the spreading of the infectious virus. Although it is difficult to declare that all the above-mentioned advances in nanobiosensors are directly applicable to real-world operations, the research published with intense efforts under urgent and evolving threats shows us what is needed to prepare for the post-COVID era and potential future crises.

## Data Availability

All datasets used in this review are included in this manuscript.

## Abbreviations

SARS-CoV: Severe acute respiratory syndrome-associated coronavirus
MERS-CoV: Middle East respiratory syndrome-related coronavirus
SARS-CoV-2: Severe acute respiratory syndrome coronavirus 2
COVID-19: Coronavirus disease 2019
WHO: World Health Organization
CDC: Centers for Disease Control
IMF: International Monetary Fund
hCoV: Human coronaviruses
RT-PCR: Reverse transcription-polymerase chain reaction
RAT: Rapid antigen tests
POC: Point-of-care
DNA: Deoxyribo nucleic acid
RNA: Ribonucleic acid
CT: Computed tomography
ELISA: Enzyme-linked immunosorbent assays
LFIA: Lateral flow immunoassays
RBD: Receptor-binding domain
ACE-2: Angiotensin converting enzyme-2
EIS: Electrochemical impedance spectroscopy
FET: Field-effect transistor
CL: Chemiluminescence
SPR: Surface plasmon resonance
LSPR: Localized surface plasmon resonance
SERS: Surface-enhanced Raman scattering
BLI: Biolayer interferometry
VTM: Viral transport medium
PBS: Phosphate buffered saline
INF: Influenza
BSA: Bovine serum albumin
HSA: Human serum albumin
IL-6: Interleukin 6
IL-8: Interleukin 8
GFP: Green fluorescent protein
LOD: Limit of detection
VOC: Variants of concern
VOI: Variants of interest
